# Murine CMV-Induced Hearing Loss Is Associated with Inner Ear Inflammation and Loss of Spiral Ganglia Neurons

**DOI:** 10.1371/journal.ppat.1004774

**Published:** 2015-04-13

**Authors:** Russell D. Bradford, Young-Gun Yoo, Mijo Golemac, Ester Pernjak Pugel, Stipan Jonjic, William J. Britt

**Affiliations:** 1 Department of Pediatrics, University of Alabama School of Medicine, Birmingham, Alabama, United States of America; 2 Department of Histology and Embryology, Faculty of Medicine, University of Rijeka, Rijeka, Croatia; 3 Department of Microbiology, University of Alabama School of Medicine, Birmingham, Alabama, United States of America; 4 Department of Neurobiology, University of Alabama School of Medicine, Birmingham, Alabama, United States of America; La Jolla Institute for Allergy and Immunology, UNITED STATES

## Abstract

Congenital human cytomegalovirus (HCMV) occurs in 0.5–1% of live births and approximately 10% of infected infants develop hearing loss. The mechanism(s) of hearing loss remain unknown. We developed a murine model of CMV induced hearing loss in which murine cytomegalovirus (MCMV) infection of newborn mice leads to hematogenous spread of virus to the inner ear, induction of inflammatory responses, and hearing loss. Characteristics of the hearing loss described in infants with congenital HCMV infection were observed including, delayed onset, progressive hearing loss, and unilateral hearing loss in this model and, these characteristics were viral inoculum dependent. Viral antigens were present in the inner ear as were CD3+ mononuclear cells in the spiral ganglion and stria vascularis. Spiral ganglion neuron density was decreased after infection, thus providing a mechanism for hearing loss. The lack of significant inner ear histopathology and persistence of inflammation in cochlea of mice with hearing loss raised the possibility that inflammation was a major component of the mechanism(s) of hearing loss in MCMV infected mice.

## Author Summary

Congenital infection with human cytomegalovirus (HCMV) is the most common viral infection of the fetus and occurs in 0.5–2.0% of all live births in most regions in the world. Infection of the fetus can result in a spectrum of end-organ disease, including long term damage to the central nervous system (CNS). Although less than 10% of infected infants exhibit clinical evidence of end-organ disease, up to 10% of the total number of infected infants develop hearing loss. Mechanisms of disease leading to hearing loss are poorly understood because of the limited availability of pathological specimens and accessibility of the inner ear. Existing small animal models fail to recapitulate many features of this infection of the inner ear. In this report we describe a mouse model in which newborn animals infected peripherally with murine CMV develop hearing loss following hematogenous spread of virus to the inner ear. Hearing loss occurs in 30–50% of animals and characteristics of hearing loss in infants with congenital HCMV infection, including delayed onset of hearing loss, progressive hearing loss, and unilateral hearing loss were present in infected mice. Our findings suggest that host derived inflammatory responses and not direct virus-mediated cytopathology are responsible for hearing loss. Findings from this study provide insight into potential mechanisms of hearing loss in infants with congenital HCMV infection.

## Introduction

Human cytomegalovirus (HCMV) is the largest human herpesviruses and an important pathogen in immunocompromised hosts [1,2]. Although the majority of adults in the world have been infected with this virus, it rarely causes an identifiable disease syndrome in normal, immunocompetent hosts. In contrast, HCMV is a frequent cause of acute and chronic disease in individuals with deficits in immunity, including the developing human fetus [1,2]. HCMV is the most common intrauterine viral infection with infection present in 0.5%-2.0% of live born infants [1]. The vast majority of infected infants exhibit no clinical symptoms at birth although severe life and organ threatening disease can be present in a small number of infected newborn infants. However, long term neurological sequelae, including abnormal auditory function can be present in infected infants regardless of the severity of disease at birth [1,3,4]. Some 10% of infants with congenital HCMV infections develop hearing loss making HCMV the most common infectious cause of hearing loss in children and accounting for approximately 25% of all cases of hearing loss in children in the US [4–6]. Studies from other areas of the world have reported similar findings [7,8, 9]. An effective vaccine to prevent this intrauterine infection is far from deployment and current antiviral agents have shown only modest benefit in congenitally infected infants [10,11].

The mechanism(s) of disease leading to hearing loss following intrauterine HCMV infection are not understood. In addition, hearing loss can develop months after the newborn period in about 50% of infected infants and be progressive in other infants, suggesting that ongoing virus replication could contribute to hearing loss [4,12]. Studies in small numbers of autopsy specimens have described HCMV in the inner ear, inflammatory cellular infiltrates, and in specimens obtained from older children, fibrosis and collagen deposits [13,14]. Guinea pigs and mice have been used as small animal models of hearing loss following HCMV infection and recapitulate some aspects of the human infection [15–22]. Although the guinea pig model is informative, the inconsistency of hearing loss and the paradox of hearing loss in non-infected guinea pigs born to infected mothers has limited interest in this model for mechanistic studies of hearing loss. In contrast, consistent infections of the inner ear structures and hearing loss can be achieved by intracochlear or intracranial inoculation of mice with murine CMV (MCMV), routes of infection that bypass the presumed viremic spread to the inner ear that follows intrauterine infection of human fetuses. Additional limitations such as the inability to model progressive hearing loss suggest that models dependent on intracranial inoculations are less reflective of the human infection and thus, have limited utility in studies of mechanisms of hearing loss that follow congenital HCMV infection. We circumvented these limitations and developed a murine model of congenital HCMV infection that utilizes intraperitoneal (ip) inoculation of newborn mice with sublethal amounts of MCMV. Shortly after infection, virus replication can be detected in the liver and spleen and during sustained viremia, central nervous system (CNS) infection is observed [23]. Because newborn mice are neurodevelopmentally equivalent to a 2^nd^ trimester human fetus, this model has allowed study of the effects of CNS infection on brain development, including auditory development that takes place postnatally in the mouse. In this report, we describe findings in this model that are consistent with the loss of spiral ganglion neurons and/or inflammation in the stria vascularis in infected mice as mechanisms of hearing loss. In addition, our results also suggested that virus-induced inflammation is likely a major component of mechanisms leading to hearing loss in MCMV infected mice.

## Results

### Cochlear infection in mice inoculated in the newborn period with MCMV

**Fig 1:**
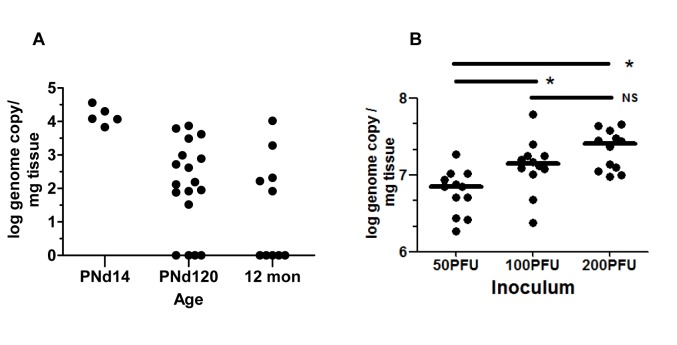
Detection of MCMV viral DNA in cochlea of mice infected as newborns with MCMV. **(A)** Newborn mice were inoculated intraperitoneally with 200PFU of Smith strain of MCMV and at the indicated times, mice were sacrificed, exhaustively perfused and cochlea removed. Viral DNA was detected using a quantitative PCR as described in Materials and Methods and expressed as log genome copies per mg of cochlear tissue. The limit of detection of this PCR assay was defined through comparison of the signal derived from DNA extracted from the cochlea of age-matched mock infected mice and was found to be less than 10 copies per mg of extracted tissue. Age post infection is shown on x-axis. On PNd14, cochlea from individual mice were pooled (n = 5) whereas at PNd120 (n = 9 mice) and 12 months (n = 5 mice), each symbol represents single cochlea. Note that results from cochlea of infected mice on PNd120 and 12 months that were below limits of detection of this assay were plotted as log = 0. The signal detected in tissue from mock infected animals was below the level of detection. **(B)** Newborn mice were inoculated with different amounts of recombinant BAC cloned Smith strain MCMV as indicated. On PNd8, cochlea (n = 12) were harvested from mice as above and the quantity of MCMV DNA (log genome copy) per mg of tissue determined as described above. Horizontal bar depicts median of total group and (*) indicates statistically significant difference in signals (50PFU vs 100PFU, p = 0.016; 50PFU vs 200PFU, p = 0.0003; 100PFU vs 200PFU, NS p = 0.215; Kruskal-Wallis test of medians with Dunn post-test correction). No signal was detected from cochlear DNA extracted from mock infected animals (n = 12 cochlea).

We previously described altered hindbrain morphogenesis in a murine model of congenital HCMV infection following infection of newborn mice with MCMV [23]. As noted above, MCMV infection of newborn mice results in hematogenous spread of MCMV to all regions of the brain [23]. Virus replication in the brain is associated with robust CNS inflammation and abnormalities in neuronal positioning in the cerebella [23,24]. These findings raised the possibility that the inner ear was also infected. Newborn mice were inoculated on postnatal day (PNd) 0 with MCMV (Smith strain) and following perfusion, cochlea were harvested on PNd14, PNd120 (4months), and 12 months post infection. Between 10^1^–10^4^ copies of MCMV DNA per mg of cochlear tissue could be detected in 100%, 77%, and 50% of cochlea at PNd14, 4 mo, and 12 mo, respectively (Fig 1A). During the course of these experiments, we initiated studies with a BAC cloned MCMV that has been shown to be essentially identical to wild type MCMV, including repair of the UL128 (mck-2) open reading frame [25]. The studies described above were repeated with this molecularly cloned virus because this BAC derived MCMV will serve as a parental virus for future studies with recombinant, mutant viruses. More relevant to the current study, inoculum effects can be studied because stocks of this virus are relatively homogenous and not a mixture of wild type and mutant viruses that has been reported to be present in uncloned Smith MCMV [25]. The BAC MCMV exhibited robust replication in newborn mice and virus could be readily demonstrated in the cochlea of infected mice on PNd8 (Fig 1B). There was an inoculum effect over a narrow range with significant differences in the recovery of viral DNA from the cochlea of mice given 50 plaque forming units (PFU) as compared to mice given 100 or 200PFU (Fig 1B). These findings demonstrated that MCMV DNA could be detected in the inner ear of newborn mice infected with MCMV and persisted in the majority of mice for over 4 months. In addition, the amount of viral DNA detected in cochlear tissue appeared to be dependent on the size of the viral inoculum.

**Fig 2:**
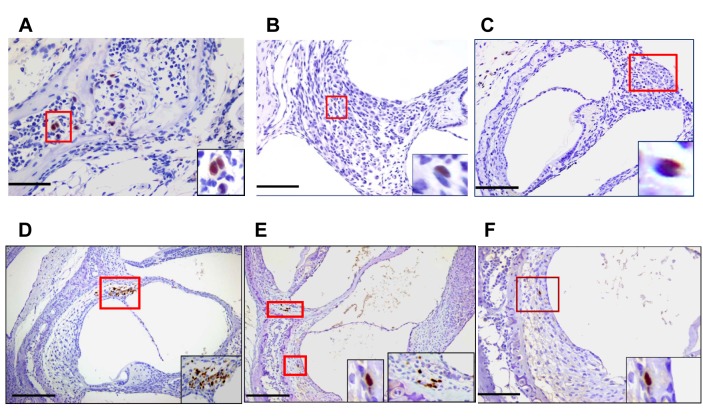
Detection of MCMV infected cells in the inner ear of MCMV infected mice. Infected cells in temporal bone marrow **(A)**, in spiral ganglion **(B,C)** and **(D-F)** in lateral wall of cochlea **(D-F)**. Panels A (x60), B,F (x40), C,D,E (x20) and boxed areas digitally zoomed in right lower corner. Panels D,E demonstrate infected cells in the spiral ligament and Panels E, F illustrate infected basal cells in the stria vascularis. Newborn mice infected with 200PFU of Smith strain MCMV were sacrificed on postnatal day (PNd) 11 and after exhaustive perfusion and fixation, the inner ears were removed, decalcified and embedded in paraffin as described in Materials and Methods. Sections were stained with anti-MCMV IE-1 (pp89) antibody CROMA 101 and visualized using a horseradish peroxidase (HRP) conjugated second antibody followed by development with diaminobenzidine (brown). Virus infected (IE-1+) cells were detected in inner ear sections from 5/8 animals examined. Scale bars indicating 50u (A), 100u (B, F,), and 200u (C,D,E) are shown at bottom left of figures.

Inner ear anatomy and histology in MCMV infected mice revealed little or no cytopathology or obvious loss of anatomical landmarks in the inner ear (S1 Fig). However, individual cells or small foci of cells expressing the MCMV IE-1 encoded protein, pp89, could be detected in the spiral ganglion, spiral ligament, stria vascularis, and in bone marrow of the temporal bones (Fig 2A–2F and S2 and S3 Figs). The distribution of virus-infected cells varied between individual animals but infected cells were most often detected in the temporal bone marrow followed by individual cells in the stria vascularis. Similar to our previous studies of infection of the brain in this model, infection was very focal with only scattered foci of infected cells or individual cells were observed in most inner ear tissue examined [23]. In contrast, virus-infected cells were not detected in the Organ of Corti and the histology of the Organ of Corti from infected mice early after infection was indistinguishable from similarly processed tissue from mock-infected mice (S1 Fig). These results together with previous results, argued that MCMV infection of newborn mice was consistent with viremic spread to the temporal bone marrow and the cochlea resulting in a focal infection of cells in the spiral ganglion, and cells in the stria vascularis and spiral ligament. These findings suggested that MCMV did not exhibit a tropism for specific cell types in the cochlea. Furthermore, infection in the inner ear did not result in significant virus-induced cytopathology within the Organ of Corti or adjacent structures in the inner ear.

### Cochlear infection in mice with MCMV results in hearing loss

To determine if cochlear infection in newborn mice resulted in hearing loss, we subjected infected and mock-infected mice to a series of auditory function tests. To eliminate the possibility that hearing loss was secondary to developmental variations in auditory function in individual animals, testing was delayed until PNd42. We initially tested mice infected with Smith MCMV virus (200pfu) and mock infected controls using 2 modalities, (1) Distortion Product Otoacoustic Emission (DPOAE), and (2) Auditory Brainstem Evoked Response (ABR). DPOAE was used to measure otoacoustic emissions from the inner ear in MCMV and mock-infected mice with DPOAE thresholds defined as the lowest stimulus intensity at which an otoacoustic emission was detectable. DPOAE thresholds were measured at 4 frequencies (4, 8, 16, and 32 kHz) and although thresholds were high for both infected and mock-infected mice, we observed an increased DPOAE threshold at only a single frequency (8 kHz) in MCMV infected mice (S4 Fig).

**Fig 3:**
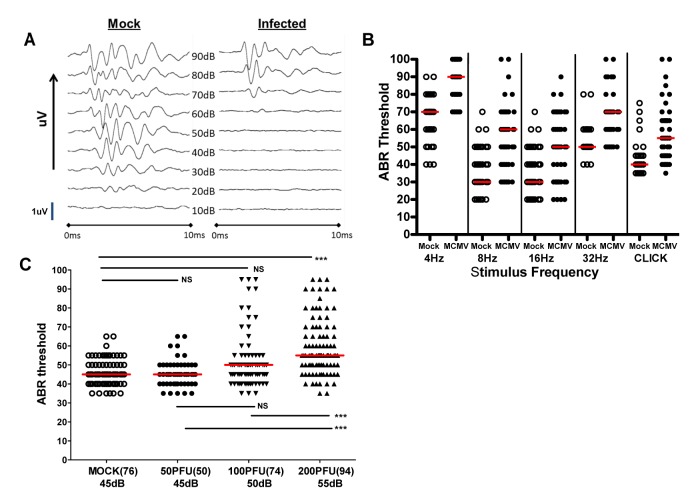
Auditory brainstem evoked response (ABR) testing in infected mice. **(A)** Example of audiogram from a MCMV infected and mock infected mouse tested over range of frequencies (click). PNd42-50 MCMV infected and mock infected mice were tested using ABR as described in Materials and Methods. These results demonstrated loss of ABR responses (ABR threshold) in the infected mouse at sound pressure level (SPL) of 60dB. Scan time over 10ms with deflection represented in microvolts (uV). The maximal amplitude of deflections varied between 0.6–1.4 uV (scale bar = 1uV) depending on the intensity of the stimulus and was derived by standard software settings established by the manufacturer (Tucker-Davis Technologies). **(B)** Pattern of ABR responses over increasing frequency of stimulus. These results demonstrate increased ABR thresholds at all frequencies tested in the mice infected with 200PFU of Smith strain of MCMV (●) and responses to click stimuli as compared to mock infected, control mice (○). There were statistically significant differences (p<0.001, Kruskal-Wallis test of medians with Dunn post-test correction) between mock (n = 25) and MCMV infected (n = 25) mice at all frequencies and with click stimulus). **(C)** Hearing loss is virus inoculum dependent. Hearing loss of mice infected with 50, 100, 200 PFU of recombinant BAC cloned MCMV. Median ABR threshold on PNd42 shown below amount of virus inoculated into newborn mice with total number of ears tested shown in parentheses. Significant differences were noted between mock infected and mice infected with 200 PFU of virus (p<0.01) and between mice infected with 50 and 100 PFU and mice infected with 200 PFU (p<0.01). Data analyzed by Kruskal-Wallis test of medians with Dunn post-test correction.

Auditory brain stem response (ABR) testing served as our standard measure of auditory function. ABR sound pressure levels (SPL) thresholds were assigned as the lowest intensity stimulus at which measurable ABR waveforms were detected as depicted in recordings from a mock and MCMV infected mouse (Fig 3A). In this experiment, ABR thresholds were absent below about 60 dB in the infected mouse whereas quantifiable responses were detected at 30 dB in the mock infected control animal (Fig 3A). Mice were tested with stimuli that included pure frequency tones at 4, 8, 16, and 32 kHz and a broadband click stimulus to establish ABR thresholds. Infected animals exhibited a significant increase in ABR threshold at every frequency of stimuli utilized as compared to mock infected animals (Fig 3B). Comparison of ABR results in mock and mice infected with 50, 100, and 200PFU of the BAC cloned MCMV revealed a statistically significant difference in the ABR thresholds between groups of mice given 200PFU as compared to mice given 50PFU and 100PFU of MCMV and mock-infected mice (Fig 3C). Although the median ABR thresholds were not statistically different in mice given 50PFU or 100PFU, the distribution of mice with thresholds above 60dB was noticeably different between these two groups, particularly in the group of mice given 100PFU. These results demonstrated that peripheral inoculation of newborn mice with two different sources of MCMV lead to altered hearing function. Perhaps more interesting was the finding that the severity of hearing loss appeared to be dependent on the size of the viral inoculum.

**Fig 4:**
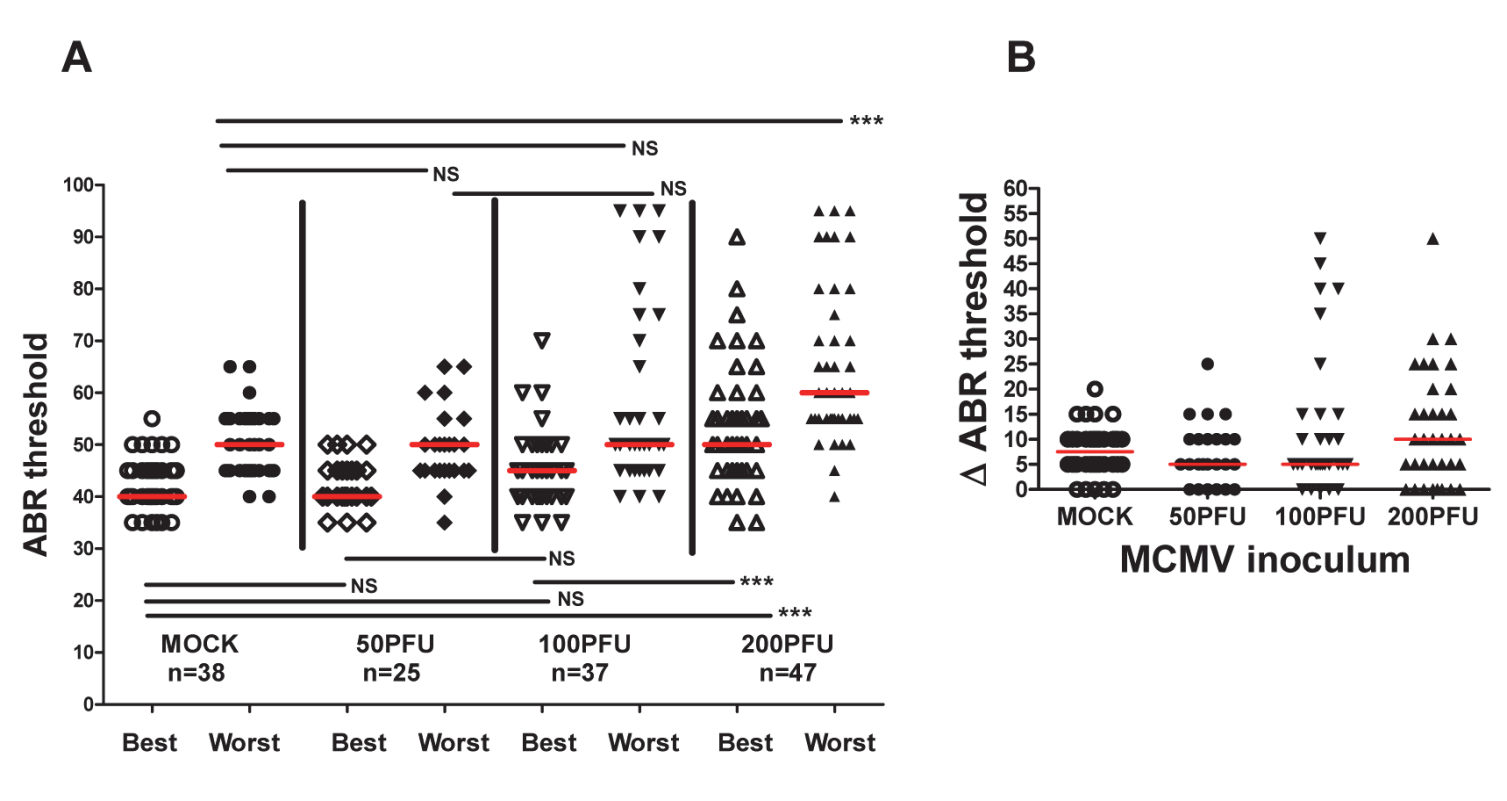
Virus inoculum dependence of ABR thresholds in individual ears of infected animals. **(A)** Medians of ABR thresholds in best and worst ears of individual mice from mock infected and mice given 50PFU, 100PFU, and 200PFU (n = number of mice analyzed). Significant differences in best ear ABR threshold between mock infected and mice infected with 50PFU and 100PFU versus mice given 200PFU and worst ears of mice infected with 200PFU versus other groups (P<0.01 by Kruskal-Wallis test of medians with Dunn post-test correction). (B) Difference (ΔABR threshold) in ABR threshold between best and worst ear. Medians of differences in ABR thresholds for best and worst plotted as function of virus inoculum. Note that 24% and 30% of mice inoculated with 100PFU and 200PFU respectively have >15dB ABR threshold difference between best and worst ear as compared to 13% and 16% for mock infected mice and mice infected with 50PFU.

In a significant number of infants congenitally infected with HCMV hearing loss can be unilateral, thus overall hearing function can be estimated by determining auditory function for the best and worst ear [4,12]. Furthermore, unilateral hearing loss is frequently observed in infants with congenital HCMV infection. To approximate such measures in MCMV infected mice, ABR thresholds were compared in the best and worst ear in mock and MCMV infected mice. This strategy allowed an additional measure of the severity of hearing loss in these groups as well as allowing us to identify animals with differences in ABR thresholds between each ear and document unilateral hearing loss in individual mice. Comparison of ABR thresholds in the best ear and worst ear of mock and mice infected with the uncloned Smith MCMV indicated that MCMV infection resulted in increased ABR thresholds in either the best or worst ear as compared to mock infected animals. To validate these preliminary findings in mice infected with the BAC derived virus, we compared ABR thresholds in mock infected and mice infected with increasing amounts of MCMV. ABR thresholds were found to be increased in the best and worst ears from mice infected with 200PFU of the BAC cloned MCMV in comparison to mock infected mice (Fig 4A). In contrast, there was no difference in the ABR thresholds between the best and worst ears of mock infected mice and mice infected with 50PFU and 100PFU of MCMV, findings that were consistent with results described above (Fig 3C). However, similar to the findings presented in Fig 3C, a sizable number of mice given 100PFU of virus had ABR thresholds >60dB as compared to mock infected or mice inoculated with 50PFU of virus (Fig 4A). When differences between ABR thresholds of the best and worst ear for individual animals were plotted as a function of MCMV inoculum, the variability in differences in auditory function between ears in individual animals was evident, particularly in mice given 100PFU and 200PFU (Fig 4B). Mice given 200PFU or 100PFU exhibited an ear-to-ear difference in ABR thresholds of at least 15dB in 30% and 24% of animals respectively as compared to 13% and 16% in mock infected and mice given 50PFU of virus. These findings demonstrated that in individual animals, severe hearing loss could be unilateral, a finding similar to the hearing loss described in infants with congenital HCMV infection [4,12]. Furthermore, an inoculum effect on hearing loss was evident as illustrated by the striking unilateral increases in ABR thresholds in individual mice, particularly in mice give 100PFU (Fig 4A). These findings raised the possibility that both host and viral functions could contributed to the disease phenotype in this model of hearing loss following viral infection.

### Progressive hearing loss occurs in mice infected with MCMV in newborn period

An important clinical feature of hearing loss associated with congenital HCMV infection is delayed onset hearing loss and/or progressive hearing deficits in a significant number of infants [4,12]. Serial ABR testing of infected and mock-infected mice during the first year of life was carried out to identify hearing loss as a function of time post-infection. Measurable declines in auditory function over time were detected in mock-infected mice as have been described in studies of age-related hearing loss in inbred strains of mice [26,27]; however, increased

**Fig 5:**
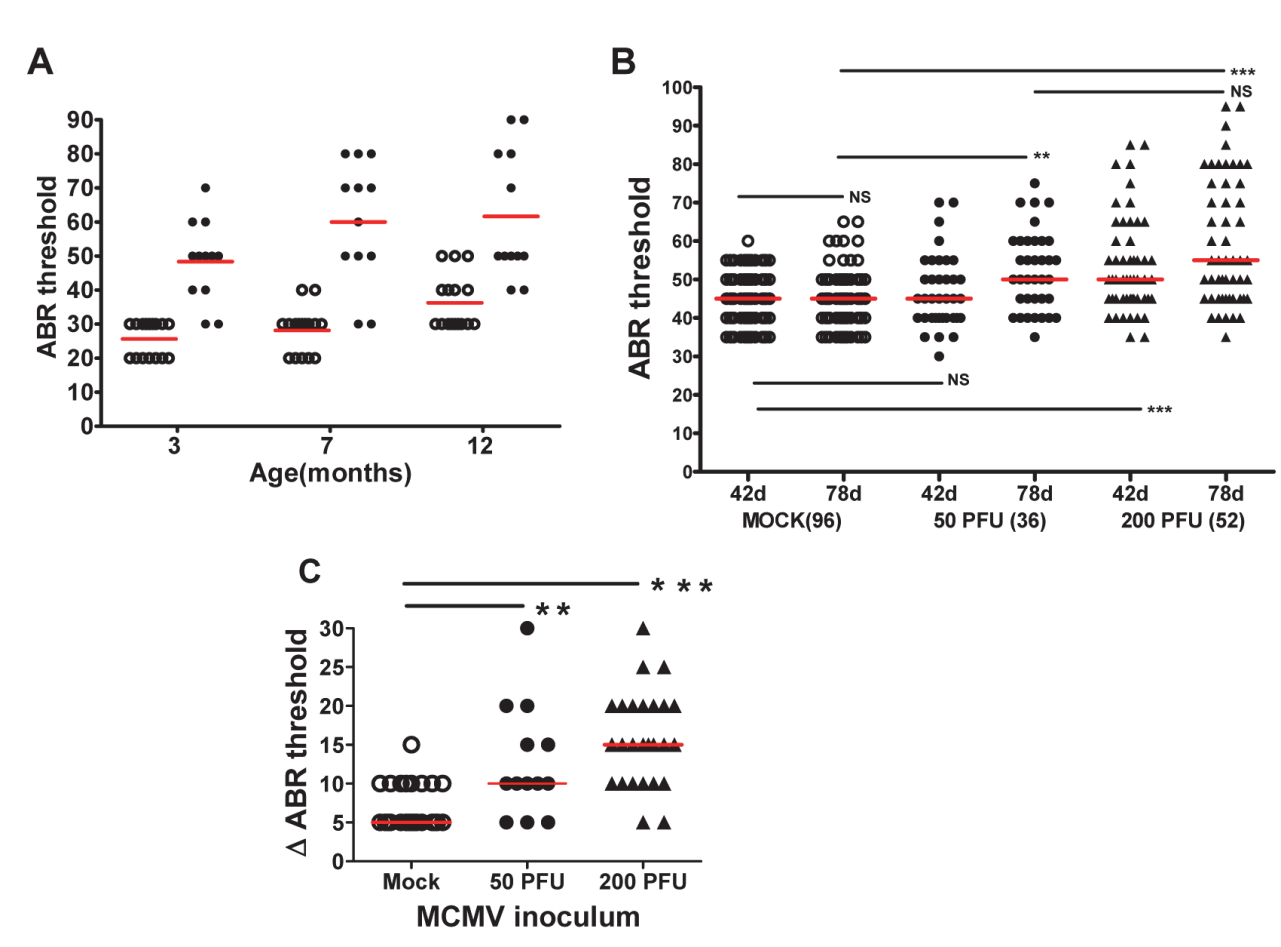
Progressive hearing loss and virus inoculum effect on progression of hearing loss in MCMV infected mice. **(A)** MCMV infected mice exhibit increased ABR threshold as function of age. Newborn mice infected with uncloned Smith MCMV(●; n = 12) or mock infected (**○;** n = 15) were tested at approximately 3,7, and 12 months and ABR thresholds determined. Note increasing mean ABR threshold in infected mice at 7 months of age and increase in ABR threshold for both infected and mock infected groups of mice at 12 months of age. At each time point the mean ABR thresholds of mock vs. infected mice were significantly different (p<0.001 two-way Anova with Bonferroni post-test correction). (**B)** Dose dependence of progressive hearing loss and delayed onset of hearing loss. Newborn mice were inoculated with recombinant, BAC cloned MCMV(50PFU ●; 200PFU ▲) or mock uninfected (**○**) and tested for auditory function at PNd42 or PNd78. Each symbol represents one ear tested (n = 96 mock, n = 36(50PFU), n = 52(200PFU)). Note the increase in median ABR threshold in mice given 50 PFU between PNd42 and PNd78 as compared to mock infected animals. (**) indicates significant differences (p<0.01) between groups with increase in ABR thresholds as determined by Kruskal-Wallis test of medians with Dunn post-test correction. **(C)** Magnitude of ABR thresholds in cochlea with progression of hearing loss. The change (dB) in individual cochlea exhibiting an increase in ABR threshold between d42 and d78 (Δ ABR threshold) was plotted for each group and medians compared. Number of cochlea with progression that were analyzed were (mock = 27; 50PFU = 13; 200PFU = 26). Statistically significant differences between median Δ ABR threshold of mock vs 50PFU, (p<0.01) and mock vs. 200PFU (p<0.001) by Kruskal-Wallis test of medians with Dunn post-test correction.

ABR thesholds were noted in about 50% of Smith MCMV-infected mice with documented hearing loss (Fig 5A). Significant increases in ABR thresholds were detected in individual mice at 3 months, 7, and 12 months of age demonstrating progressive hearing loss in infected mice (Fig 5A). Progression of hearing loss was observed between 3 and 7 months of age with 4 of 9 mice exhibiting an increase in ABR thresholds (Fig 5A). However, there was no further increase in the number of mice with increased ABR thresholds between 7–12 months of age suggesting that auditory function stabilized in these animals 7 months post infection, a finding that recapitulates the natural history of hearing loss in later childhood in children with congenital HCMV infection [4,12]. Finally, several mice in both the mock and MCMV infected groups demonstrated a modest improvement (<10dB) in hearing with a decline in ABR thresholds between 7 and 12 months (Fig 5A). Although we have no explanation for this finding at this time, fluctuating hearing loss is a well described audiologic finding in infants with congenital HCMV infection [4,12].

To investigate the influence of virus inoculum on progressive hearing loss, hearing was analyzed as a function of time post infection in mice given 50 and 200PFU of BAC cloned MCMV. There was an increase in ABR thresholds between PNd42 and 78 in mice given both 50PFU and 200PFU as compared to mock infected mice (Fig 5B). Importantly, mice inoculated with 50PFU of virus had similar ABR thresholds as mock infected mice on PNd42, but as a group, the median ABR threshold of mice infected with 50PFU increased to approximately 50dB when tested at PNd78, a difference that was significantly higher than age-matched mock infected mice (Fig 5B). The magnitude of the increase in ABR thresholds was analyzed by plotting difference in ABR thresholds on PNd42 and 78 in only individual ears that exhibited increased ABR thresholds (Fig 5C). The difference in median ABR thresholds over this interval was 5dB for mock-infected animals as compared to 10dB and 15dB for ears from animals given 50 and 200PFU of MCMV, respectively (Fig 5C). Interestingly, in individual infected animals, very significant increases in the ABR thresholds were observed (Fig 5C). Thus, these results utilizing a different source of virus confirmed that progressive hearing loss developed in MCMV infected newborn mice. Perhaps of more interest was the finding of delayed onset of hearing loss in mice given a virus inoculum that failed to produce hearing loss at initial testing on PNd42. This finding paralleled observations in human infants congenitally infected with HCMV in which up to 50% of infants who ultimately develop hearing loss will exhibit normal hearing early in infancy [4,12].

**Fig 6:**
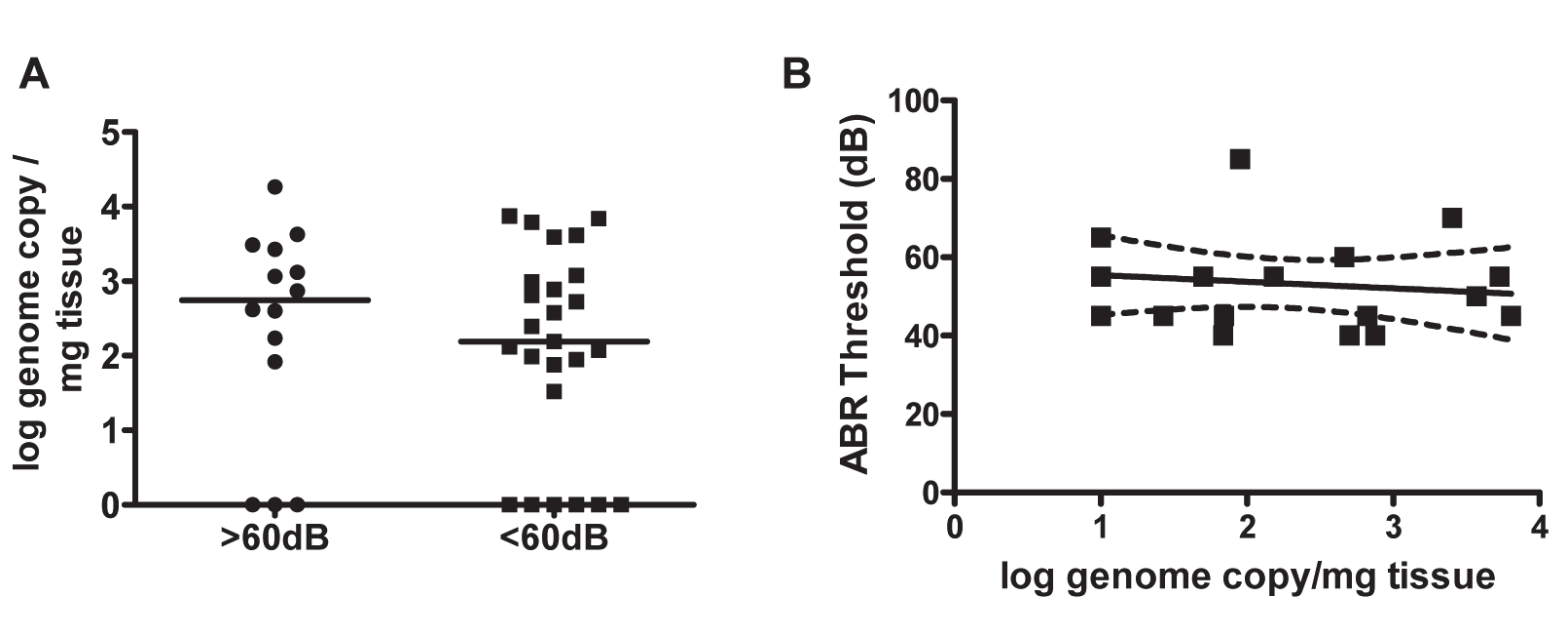
Hearing loss in MCMV infected mice is not correlated with virus load in the cochlea. **(A)** MCMV log genome copies/mg tissue in cochlea from mice with ABR thresholds >60dB (median 71dB; n = 14) and ABR thresholds <60db (median 45dB; n = 25) at PNd42. Groups did not differ in quantity of viral DNA in cochlea (Mann-Whitney, p = 0.42). Note that results from some cochlea were below limits of detection of PCR and therefore indistinguishable from mock infected cochlea. These were plotted as log = 0 to permit statistical comparison of groups. **(B)** Linear regression analysis of ABR thresholds versus genome copies in cochlea (n = 18 mice). There was no correlation between viral genome copy number and hearing (p = 0.59, slope not significant from 0).

The findings that, (i) virus persisted in the inner ear for over four months (Fig 1), (ii) an increase in viral inoculum was associated with an increase ABR thresholds (Fig 3) and, (iii) an increase in viral inoculum was correlated with progression of hearing loss (Fig 5) suggested that the quantity of virus in the inner was directly related to hearing loss in this model. We explored this possibility by comparing the quantity of viral DNA in the cochlea of infected mice with ABR thresholds >60dB to mice with ABR thresholds <60dB. There was no significant difference in the quantity of cochlear viral DNA in cochlea from mice with ABR thresholds >60dB (median 71dB) or <60dB (median 45dB) (Fig 6). These results argued that although there was a clear relationship between the size of the initial viral inoculum and development of an increased ABR threshold (and progression), there was not a direct relationship between cochlear viral load and increased ABR thresholds when tested at PNd42.

### Cochlear infection in MCMV infected mice is associated with decreased cellular density in spiral ganglia

**Fig 7:**
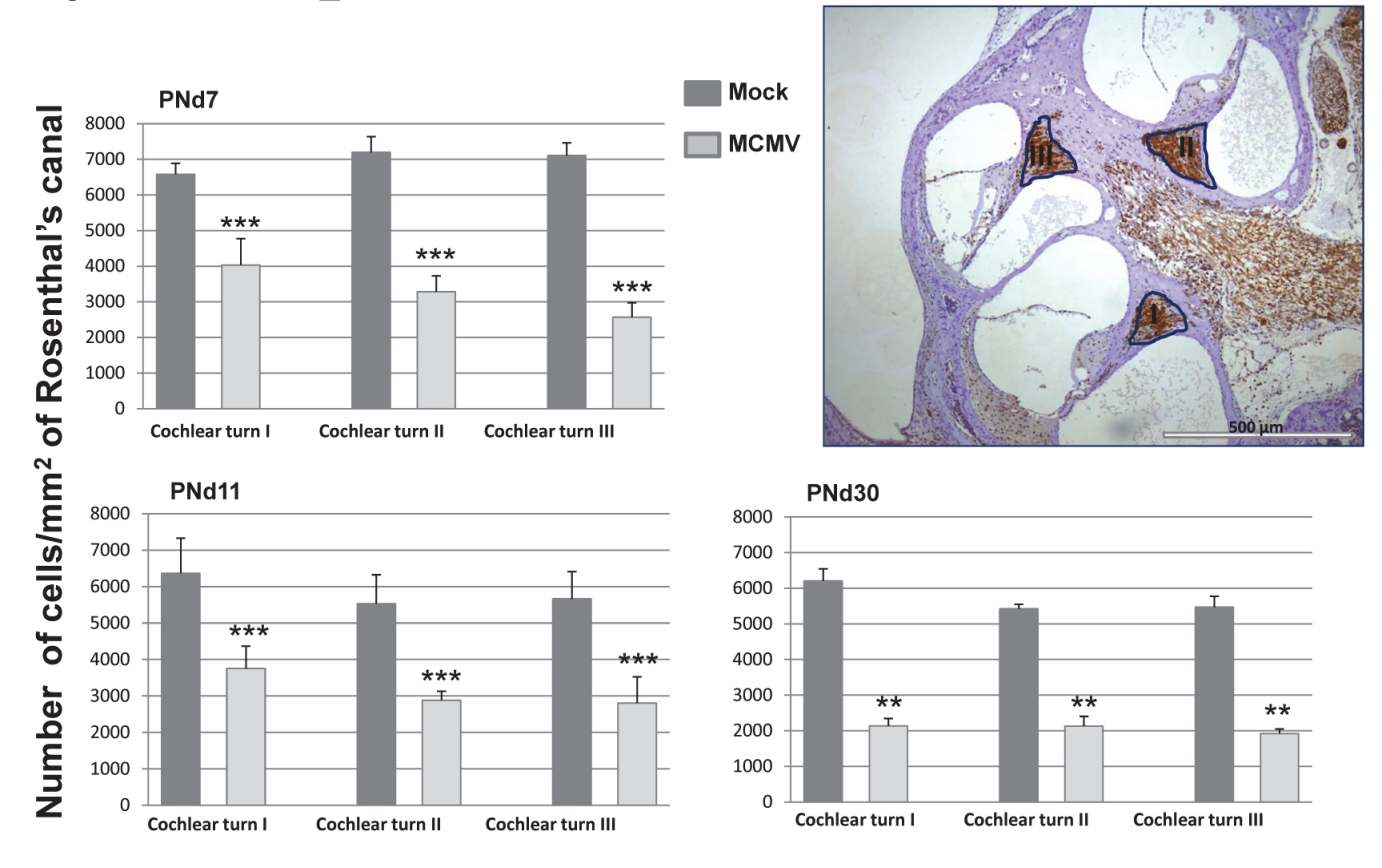
Loss of spiral ganglion neurons in mice infected in newborn period with MCMV. Mice were sacrificed at various postnatal days (PNd) 7, 11, and 30 as described in the Material and Methods. For each time point, cochlea were removed and placed in 4% paraformaldehyde, decalcified and embedded in paraffin as described in Materials and Methods. Sections were stained with anti-Tuji 1 antibodies to detect neurons followed by horseradish peroxidase (HRP) conjugated second antibody and development with diaminobenzidine (brown). Cresyl violet (CV) was used as counterstain. Digitalized images, of the inner ear from each individual tsection (2 per cochlea) were imported into the *B-Cell Image* program. Spiral ganglion cell (SGC) densities were calculated in infected animals (4 mice per time point) and mock infected animals (3 mice per time point) by determining the number of spiral ganglion neurons followed by normalization to the area of Rosenthal’s canal in each section to provide the spiral ganglion neuron density (cells/mm^2^). Spiral ganglion neuron densities in the basal, mid and apical turns (I, II, III) of the cochlea are presented as the mean number of neuron density with error bars demarcating SEM (** indicates significant differences p<0.0022, and *** p<0.001 by Mann-Whitney).

To determine if further histologic analysis of the inner ear in mice with increased ABR thresholds could point to mechanisms of disease in the auditory system, cochlear tissue from a group of mock and MCMV infected animals with and without hearing loss were analyzed. No obvious differences in cochlear histology were found between mock infected, infected mice with normal hearing, and infected mice with abnormal hearing (S5 Fig). Because a correlation between the loss of spiral ganglion neurons loss increased ABR thresholds has been previously described in other rodent models of hearing loss, we determined spiral ganglion neuron density in MCMV infected mice [28–30]. Enumeration of the number of Tuji-1 positive, nucleated cells in defined areas of the spiral ganglia in infected and mock infected mice indicated that spiral ganglia from MCMV infected animals contained a lower density of Tuji-1+ cells as compared to age matched, mock infected mice at three different time points (Fig 7 and S6 Fig). These results suggested that increased ABR thresholds in MCMV infected mice could be explained in part, by the loss of neurons in the spiral ganglia.

### Hearing loss in MCMC infected mice is associated with persistent expression of proinflammatory responses in the cochlea

**Fig 8:**
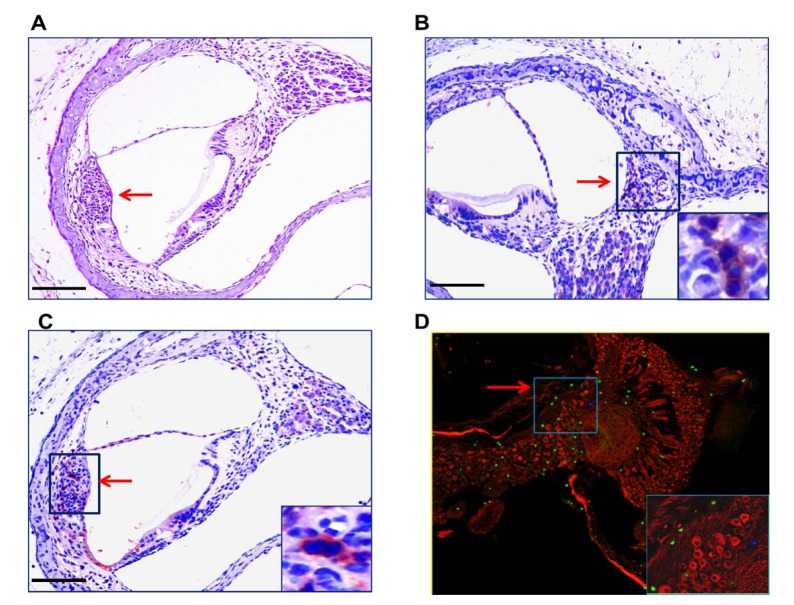
Mononuclear cells infiltrates in the cochlea of infected mice. **(A)** Mononuclear cell infiltrate in the cochlea of infected mice. H&E staining of cochlea demonstrates mononuclear cellular infiltrates in the stria vascularis (arrow). **(B,C)** Immunohistochemical detection of CD3+ mononuclear cells in spiral ganglion (B) and stria vascularis(C) (20x). Cochlea from infected mice were harvested a PNd11 and following extensive perfusion, cochlea were fixed and sections for immunohistochemistry as described in Materials and Methods. Staining with anti-CD3 antibodies, detection with a HRP conjugated secondary antibody and development with AEC was carried as described in Materials and Methods. Arrows depict CD3+ cells with digital zoom boxed in lower right corner an scale bars 200um in left corner. **(D)** Detection of CD3+ mononuclear cells in spiral ganglion of MCMV infected mouse with hearing loss. Frozen section from the cochlea from PNd120 mouse with hearing loss documented on PNd50 was reacted with rabbit anti-CD3 and mouse anti-Tuj1 antibodies, followed by staining with FITC anti-rabbit IgG secondary antibodies or tetramethylrhodamine isothiocyanate conjugated anti-mouse IgG secondary antibodies and viewed under confocal microscope (40x). Note CD3+ cells (green) and Tuj1 positive cells (red) neurons in boxed area (60x) in lower right hand corner.

Even though the development of hearing loss in MCMV infected mice was related to the magnitude of the intial viral inoculum, the lack of a direct relationship between cochlear viral DNA and increased ABR thresholds raised the possibility that MCMV-induced inflammation and not direct viral cytopathic effects, could be responsible for hearing loss in MCMV infected mice. This mechanism was also suggested by both limited cytopathology in the cochlea (S1 Fig) and the focal nature of the virus infection within the cochlea (Fig 2). We initially explored this possibility by assaying mononuclear infiltration into the cochlea in infected mice. A mononuclear cellular infiltrate, including CD3+ mononuclear cells, could be detected early after in infection into both the stria vascularis and the spiral ganglion (Fig 8A, 8B and 8C). In addition, CD3+ mononuclear cells were detected in the spiral ganglion of infected mice with hearing loss on PNd120 of life (Fig 8D). In a second analysis, increased transcription of multiple proinflammatory molecules was detected in cochlear tissue from infected mice as early as PNd4, increasing by PNd8, and in some mice, declining by PNd16 (S1 Table). Interestingly, the expression of a subset of proinflammatory chemokines that contribute to mononuclear cells infiltration into virus infected tissue appeared associated with the size of the virus inoculum, a finding that paralleled the increased rate of increased ABR thresholds in mice given larger virus inoculums (Fig 3 and Table 1). The relationship between inflammatory responses in the cochlea of infected mice and hearing loss was further explored in groups of mice with and without increased ABR thresholds when initially tested on PNd42 (Table 2). When we assayed the level of expression of this set of proinflammatory molecules in cochlear tissue from mice with ABR thresholds >60dB (median ABR threshold 75dB) and those with ABR thresholds <60dB (median ABR threshold 45dB), we noted that that several chemokines, including CCL8, CXCL9, and CXCL10, with potent chemoattractant activities for cells contributing to tissue inflammation remained persistently elevated in the group of animals with ABR thresholds >60dB (Table 2). These findings together with the lack of significant inner-ear cytopathology raised the possibility that host derived inflammatory responses in the cochlea of infected mice likely played a critical role in hearing loss in this model of virus-induced hearing loss.

**Table 1:** Levels of expression of proinflammatory chemokines in cochlea of infected mice are dependent on viral inoculum.

Inoculum (PFU)						
	50 (n = 12)		100 (n = 12)		200 (n = 12)	
Chemokine	Fold Change	p value	Fold Change	p value	Fold Change	p value
**CCL1**	**33.0**	**<0.001**	**33.3**	**<0.00005**	**82.5**	**<0.01**
**CCL3**	**2.17**	**<0.0005**	**5.52**	**<0.00005**	**13.3**	**<0.0001**
**CCL4**	**11.3**	**<0.0001**	**26.7**	**<0.00005**	**64.1**	**<0.00001**
**CCL5**	**5.70**	**<0.00001**	**20.4**	**<0.00001**	**52.3**	**<0.00001**
**CCL8**	**164**	**<0.00005**	**63.5**	**<0.00001**	**60.7**	**<0.05**
**CXCL9**	**276**	**<0.00001**	**754**	**<0.00005**	**1328**	**<0.00001**
**CXCL10**	**168**	**<0.00001**	**323**	**<0.00001**	**911**	**<0.00001**
**CXCL11**	**8.49**	**<0.00001**	**15.0**	**<0.001**	**39.0**	**<0.00001**

Cochlear tissue isolated from infected and uninfected, control mice on PNd8 with number of mice in each indicated. Expression of individual genes determined as described in Materials and Methods and reported as fold change and statistical comparison with control group (p value). Note increasing fold change with increasing viral inoculum.

**Table 2:** Persistent expression of proinflammatory chemokines in cochlea of mice with ABR thresholds >60dB.

	**PNd10**		**PNd42**			
			ABR <60 dB		ABR>60 dB	
**Chemokine**	Fold Change	p value	Fold Change	p value	Fold Change	p value
CCL1	**32.4**	**0.002**	**5.21**	**0.002**	**5.95**	**0.03**
CCL2	**17.1**	**0.00002**	**1.49**	**0.050**	**3.36**	**0.15**
CCL4	**17.4**	**0.00008**	**1.21**	**0.28**	**1.69**	**0.31**
CCL5	**19.2**	**0.0002**	**1.26**	**0.41**	**1.36**	**0.22**
CCL7	**16.4**	**0.001**	**1.53**	**0.27**	**3.53**	**0.15**
CCL8	**39.0**	**0.002**	**3.47**	**0.19**	**6.62**	**0.0007**
CXCL9	**592**	**0.000018**	**2.23**	**0.17**	**3.22**	**0.026**
CXC10	**395**	**0.0005**	**2.54**	**0.14**	**2.95**	**0.024**
CXCL11	**101**	**0.0003**	**3.56**	**0.07**	**5.42**	**0.08**

Mice were infected with MCMV or mock infected as controls as described in Materials and Methods. One group of infected mice (n = 12) and control mice (n = 6) were harvested at PNd10. A second group underwent ABR testing on PNd42 and equal numbers (n = 12) of mice with ABR thresholds >60dB (median 75dB) or <60dB (median 45dB) were harvested, cochlea pooled from individual mice and expression of proinflammatory chemokines determined as described in Materials and Methods. Results are reported as fold change and statistical comparison to age matched control mice (p value). Note increased expression of CCL8, CXCL9, and CXCL10 in mice with hearing loss.

## Discussion

Although the association of hearing loss with congenital HCMV infection has been recognized for over 4 decades, the pathogenesis of this sequelae of congenital HCMV infection remains poorly understood. Early studies of temporal bones from a small number of infants and older children with congenital HCMV infections described viral antigens and viral particles in the inner ear, inflammatory cellular infiltrates in the inner ear, and degeneration, calcifications, and fibrosis of structures in the inner ear in older children [31]. More recently an informative study of tissues from 6 fetuses terminated between 19–35 weeks of gestation secondary to intrauterine HCMV infection described viral antigens and inflammatory infiltrates consisting of CD3+ mononuclear cells and macrophages in the inner ears [32]. Within the cochlea, 5/6 fetuses had detectable viral inclusions in the stria vascularis associated with mononuclear cells in proximity to infected cells [32]. Neither viral nucleic acids nor viral antigens were detected in the spiral ganglia but mononuclear cells were present in the spiral ganglia/VIIIth nerve in 4/6 of fetuses [32]. Extensive autolysis in the Organ of Corti limited description of this region of cochlea [32]. In the current study, we have described findings that were remarkably similar to this report, including scattered virus infected cells in the spiral ganglia and stria vascularis as well as a cellular infiltrate of mononuclear cells. There was no significant virus-induced cytopathology or tissue destruction in the cochlea, including in the Organ of Corti; however, our analysis was limited in that it did not include studies of whole cochlear sections that could provide an estimate of hair cell number. We documented hearing loss as measured by increased ABR thresholds in 20–50% of mice inoculated with MCMV during the newborn period suggesting that the histopathologic findings that we observed were associated with impaired auditory function.

Deficits in hearing in MCMV infected mice were documented primarily by findings of increased ABR thresholds. Although our findings in DPOAE testing suggested normal hair cell function in infected animals, elevated stimulus thresholds in our DPOAE protocol could have obscured abnormal test results. However, the lack of cytopathology in the Organ of Corti, increased ABR thresholds, and the loss of spiral ganglion neurons suggested that hearing loss in this model was consistent with lesions in the auditory system other than hair cell loss. In contrast to our results, two recent studies utilizing intracranial (IC) inoculation of newborn mice with MCMV to produce hearing loss documented hair cell loss [18,19]. These investigators also reported that IC inoculation lead to infection of spiral ganglion neurons but not in cells within the Organ of Corti, including hair cells [19]. Mononuclear cellular infiltrates in the cochlea of IC infected animals suggested that hair cell and hearing loss in this model was secondary to virus-induced inflammatory responses [19]. Although informative, IC inoculation in these models also eliminates major characteristics of CNS infection following intrauterine HCMV infection including physiologic routes of virus spread to the CNS and the inner ear, infection of brain endothelium, and CNS recruitment of inflammatory cells from peripheral sites of infection. Thus, spread of MCMV to the inner ear structures following IC inoculation is almost certainly different than hematogenous routes that occur in the infected human fetus. It has been argued that hematogenous spread to the inner ear of mice inoculated intraperitoneally could be differentiated from spread along the cochlear nerves (meninges) and from the ventricles that occurs in IC inoculated animals [21].

Other small animal models of CMV associated hearing loss have been reported, including congenital infections in guinea pigs (gpCMV). Although this model includes hematogenous spread of virus to the inner ear, it is also associated with modest and often inconsistent inner ear infection, hearing loss in animals without inner ear infection, and confounding issues associated with the severity of maternal infection required to reproducibly achieve intrauterine transmission [33]. This latter characteristic of this model has raised the possibility that placental damage may underlie major manifestations of disease in newborn pups. Murine models that permit infection of the developing mouse embryo have been described, including direct injection of MCMV into the placenta in mid-gestation [20]. In this model hearing loss was achieved and viral nucleic acids could be detected in the inner ear; however, a direct relationship between hearing loss and virus in the cochlea was not described and no evidence of inflammation was found [20]. In contrast to these previously described small animal models of CMV associated hearing loss, the model described in this manuscript recapitulates many key characteristics of hearing loss associated with congenital HCMV infections including; (i) virus infection and virus-induced inflammation during the development of auditory pathways, (ii) physiologic spread of this virus to the CNS and inner ear and, (iii) similar heterogeneity in the phenotypes of hearing loss observed in infants with congenital CMV infections, including unilateral loss, delayed onset and progressive hearing loss [4, 12].

Larger initial inoculums of MCMV resulted in increased amounts of virus in the inner ear and an increase in the incidence of hearing loss suggesting a direct relationship between virus replication in the cochlea and hearing loss; however, by PNd 42 there was no correlation between virus in the cochlea and hearing loss suggesting that persistent virus replication in the cochlea was not directly related to hearing loss. Thus, it could be argued that only the quantity of virus present in the cochlea early in infection was related to the development of hearing loss. Possible explanations that could account for these findings include the increased likelihood of infection of specific cell types in the inner ear following infection with larger inoculums or, alternatively, inoculum dependent induction of cochlear inflammation early in infection that was not reflected in levels of virus replication at later time points. Thus, larger virus inoculums resulted in increased virus replication and as we observed, a corresponding increase in expression of some proinflammatory cytokines and chemokines early in infection (Table 1). These early events could be responsible for the increase in the incidence of increased ABR thresholds in mice given larger amounts of virus. Consistent with this mechanism was that in later stages of infection when hearing function could be determined, mice with hearing loss had persistent cochlear expression of a subset of proinflammatory chemokines. The lack of widespread histopathologic damage in the cochlea of MCMV infected mice and the focal nature of the inner ear infection together with the robust expression of proinflammatory molecules argued that cochlear inflammation could represent a proximal cause of hearing loss in infected mice. Lastly, the mechanism(s) of inner ear damage leading to delayed onset and/or progressive hearing loss remain explained. However, as noted below, damage to the auditory pathways within the developing inner ear can result in ongoing loss of spiral ganglion neurons long after the initial insult [34].

Observations in other small animal models of hearing loss as well as findings in human temporal bones are consistent with a mechanism(s) of virus-induced inflammation; however, specific lesions and the pathways leading to hearing deficits have not been well described. Virus infected cells and mononuclear cells in the spiral ganglion are common to rodent models as well as tissue from infected fetuses, findings suggesting that disruption of spiral ganglion function could contribute to hearing deficits in MCMV infected mice. The loss of spiral ganglion neurons following MCMV infection in our model are similar to findings reported in a more limited analysis of a murine model of CMV associated hearing loss [20]. Thus, loss of spiral ganglion neurons could contribute to a common mechanism of hearing loss that follows cochlear infections with CMV and perhaps, parallels the proposed pathogenesis of hearing loss associated with bacterial meningitis.

A decrease in the number of spiral ganglion neurons provided a potential mechanism of hearing loss in infected animals secondary to loss of afferent input into cochlear nuclei during this critical time of auditory development [35,36]. Studies have documented neuronal loss in cochlear nuclei in the brainstem associated with loss of afferent input into the cochlear nuclei during the so called critical period prior to the onset of hearing in mammals [37–39]. This time period between PNd5-11 has been described as the critical period in mice because ablation of the cochlea after PNd14 does not result in loss of neurons in the cochlear nuclei secondary to the acquisition of survival (anti-apoptotic) functions in neurons within the cochlear nuclei [40,41]. A similar pro-apoptotic program of spiral ganglion neurons has been described in rodents with aminoglycoside-mediated damage to hair cell function [34]. Interestingly, apoptosis of spiral ganglion neurons in this model was prolonged with spiral ganglion neurons loss occurring over a 14 week period, a finding that could explain the progression and delayed onset of hearing loss in our model of hearing loss [34]. It should be stressed that our data cannot directly link spiral ganglion loss with virus infection/virus-induced inflammation because damage to hair cells in the Organ of Corti or altered function of the stria vascularis epithelium could also lead to loss of spiral ganglion neurons or impaired electrical output from these cells. Finally, survival and neurite extension of spiral ganglion neurons is dependent on afferent input and on expression of the neurotrophins, BDNF and NT3 [42–45]. Previously, we documented decreased expression of phospho-TrkB (NTRK2), the high affinity receptor tyrosine kinase for BDNF, in cerebella from MCMV infected mice during early postnatal period (7–10 PN d of age) raising the interesting possibility that decreased signaling through TrkB could have also contributed to spiral ganglion neuron loss in MCMV infected mice [23].

The finding of virus infected cells and CD3+ mononuclear infiltrates in the stria vascularis suggested additional mechanisms of hearing loss in MCMV infected mice. This region of the inner ear is critical for maintaining the unique electrolyte composition of the extracellular fluid within the Organ of Corti that contributes to electrical potential of the endocochlear fluid, the endocochlear potential [46,47]. Hair cells within the Organ of Corti exhibit spontaneous action potentials during early postnatal life required for development of hearing pathways, including afferent input into neurons in the spiral ganglion [48,49]. The inflammatory cellular infiltrate as well as virus in this region of the cochlea raised the possibility that altered function of this specialized epithelium could in turn, lead to deficits in hearing development in infected mice. Interestingly, mutations in the gene encoding connexin 26 and 30, components of gap junctions within the stria vascularis that are essential for maintenance of the endocochlear potential, represent two of the most common genetic mutations associated with hearing loss [46].

In summary, we have described a model of hearing loss in mice inoculated early in the postnatal period with MCMV that closely recapitulates key aspects of hearing loss associated with congenital HCMV infection. Our studies have documented the dependence of viral replication in the cochlea on the initial viral inoculum size and in turn, the level of expression of proinflammatory molecules in the cochlea as well subsequent increases in the ABR thresholds on the level of virus replication during this early time in auditory development. Although virus infected cells could be detected in various regions of the inner ear, significant cytopathology or tissue damage was absent arguing that the mechanism(s) of hearing loss following this infection could have a significant inflammatory component.

## Materials and Methods

### Ethics statement

The University of Alabama-Birmingham Institutional Animal Care and Use Committee (IACUC) guidelines were followed for all animal experiments and breeding. These guidelines are in strict compliance with the NIH (OLAW Assurance Number—A32555-01) guidelines. Animals were euthanized with carbon dioxide under regulated flow. Following carbon dioxide, cervical dislocation was implemented to ensure animals were completely euthanized prior to carrying out experimental protocols, according to the UAB Animal Resource Program (ARP) guidelines. An approved protocol (APN09024) was obtained from IACUC (UAB) for all experiments involving animals and animal tissues carried out at UAB. All procedures involving animals and their care conducted at the Faculty of Medicine, University of Rijeka were approved by the Veterinary Department of the Ministry of Agriculture, Croatia (protocol class UP/I-322-01/13-01/32; number 525-10/0255-13-2 for the NIH grant R01 NS065845; protocol class UP/I-322-01/13-01/33; number 525-10/0255-13-2 for the NIH grant R01 AI089956 and the Office of Laboratory Animal Welfare (OLAW)—Assurance no. A5905-01 (effective until 10/31/2019) and were conducted in compliance with institutional guidelines, as well as with national (Animal Protection Act 135/06, 37/13, 125/13; Regulations on the Protection of Animals Used for Scientific Purposes 55/2013) and international Directive 2010/63/EU of The European Parliament and of the Council of 22 September 2010 on the protection of animals used for scientific purposes, Guide for the Care and Use of Laboratory Animals, National Institutes of Health, The National Academies Press, Washington, DC) laws and policies.

### Mice

Pathogen-free BALB/c mice were obtained from a colony propagated from animals purchased from Charles River Laboratories. All breeding and experiments were done in accordance with the guidelines of the University of Alabama at Birmingham Institutional Animal Care and Use Committee and with the University of Rijeka-Croatia animal use policies. Auditory testing including ABR and DPOAE (see below) measurements were performed in anesthetized animals (ketamine (75 mg/kg) and medetomidine (1 mg/kg) delivered i.p.). Animals were maintained at 37°C during all testing and monitored for movement.

### Virus and infection

Virus stocks (Smith strain of MCMV, ATCC VR-194, American Type Tissue Culture (ATCC)) were propagated in mouse embryonic fibroblasts. In some experiments, a virus that has been determined by sequence analysis and characterization in-vivo to be wild type Smith virus was recovered from an infectious BAC clone of MCMV kindly provided by Dr. Ulrich Koszinowski (University of Munich) [25]. Virus recovered from the MCMV BAC was sequenced to insure that the previously described mutation that resulted in truncation of the UL128 open ready frame had been repaired to wild type sequence [25]. Infectious virus was produced by passage through mouse bone marrow cells (MBN1084, ATCC) as described [25]. Newborn pups (6–18 h postpartum) were inoculated intraperitoneally (i.p.) with 200 PFU of tissue culture passaged MCMV Smith strain and between 50-200PFU of virus recovered from the BAC as described [24].

### Distortion Product Otoacoustic Emission (DPOAE)

DPOAEs were measured using Tucker Davis Technologies (TDT, Alachua, FL) System III equipment and software using published protocols [50]. Stimuli were generated at 4, 8, 16, and 32 kHz with the fixed ratio of frequency 2 (f2) to frequency 1 (f1) of 1.22 and delivered to the ear via closed-field electrostatic speakers (EC1) connected to a coupler housing a low noise microphone (ER10B+, Etymotic, Elk Grove Village, IL), whose output was processed by an MA3 microphone amplifier. The 2f1-f2 otoacoustic emission was measured, and DPOAE threshold was determined as the lowest intensity at which the 2f1-f2 response was measurable above the noise floor.

### Auditory Brainstem Evoked Response (ABR)

ABRs were also measured with the TDT System III system, using tone pips at 4, 8, 16, and 32 kHz (90 dB to 20 dB in -10 dB steps), as well as broadband click stimuli (90 dB to 20 dB in -5 dB steps). Signal was detected using 3 stainless steel needle electrodes (vertex, ipsilateral ear, low back), amplified, band-pass filtered (300 Hz high-pass, 3 kHz low-pass, 60 Hz notch), and averaged over 750 sweeps. The ABR threshold was defined as the lowest intensity signal at which an ABR waveform was detectable. We adapted protocols from several sources including material provided during a training course in audiometric testing at Jackson Labs (Bar Harbor, Ma) and published protocols [51].

### Histology

Animals were exhaustively perfused initially with phosphate buffered saline (pH 7.4; PBS) and then with 4% paraformaldehyde (PFA) as described [24]. Cochleae were dissected away from the skull and were soaked in cold PFA for ∼6 h, followed by three 10 min PBS washes. Samples were decalcified in 120 mM EDTA, changed daily x 5 days. After decalcification, samples were soaked until they sunk to the bottom of the container in 30% sucrose as a cryoprotectant during the freezing in preparation for cutting of 8-um frozen sections [24]. After fixation, some cochleae from infected and noninfected animals were embedded in paraffin for sectioning. 4-μm mid-modiolar sections were cut and processed for H&E or Cresyl violet staining and/or immunohistochemistry. In most cases, 50 sections were cut from individual ears for H&E(Cresyl violet) staining and for IHC (every 10^th^ section). Immunohistochemical (IHC) staining for the IE-1 protein (pp89) encoded by MCMV IE-1 was performed as described previously [23]. Anti-CD3 staining was performed using a rabbit anti-mouse CD3 antibody (Abnova, AB5690) and developed with a horseradish peroxidase (hrp)-conjugated second antibody followed by aminoethylcarbazole (AEC;Sigma, St.Louis, Mo). For neuron staining, neuronal class IIIβ-Tubulin (TUJ1) rabbit monoclonal antibody (MRB-435P COVANCE) were used followed by goat anti-rabbit IgG H&L (Biotin) (Ab6720) and developed with DAB chromogen (Dako). Frozen sections stained with appropriate antibodies were viewed under confocal microscope [24].

### Quantitative PCR for viral DNA and RNA and PCR array for antiviral and proinflammatory molecule quantification

Nucleic acids were isolated from cochleae using several protocols depending on downstream applications. In the initial experiments, nucleic acids were isolated using a QIAGEN M48 robot and MagAttract RNA Tissue Mini M48 kit as per the manufacturer’s instructions (Qiagen, Valencia, CA). In latter experiments, RNA and DNA nucleic acids were isolated using SQ Total RNA Kit and HP Total RNA Isolation Kit (Omega Bio-Tek, Norcross, GA) with minor modifications. Quantitative real-time PCR was performed by amplification of a fragment of immediate-early 1 (IE1) exon 4 using the primers forward, 5'-GGC TCC ATG ATC CAC CCT GTT A-3' and reverse, 5'-GCC TTC ATC TGC TGC CAT ACT-3'. The probe (5'-AGC CTT TCC TGG ATG CCA GGT CTC A-3') was labeled with reporter dye FAM and quencher dye TAMRA. To quantify viral genome copy number, serial dilutions (log10) of a plasmid with the cloned IE1 exon 4 PCR product was used to create a standard curve. This assay has a sensitivity of between 50–100 copies of MCMV DNA. For the detection of inflammatory network genes, the Mouse Antiviral Response PCR Array (PAMM-122A; Qiagen, Valencia, CA) and for proinflammatory chemokines and cytokines (PAMM-150Z) were used according to manufacturer's protocol. Quantitative real-time PCR was conducted using TaqMan Gene Expression master mix (Qiagen). Samples were run in triplicate using a two-step amplification protocol on the Applied Biosystems 7500 Real-Time PCR System (Applied Biosystems, Life Technologies.

## Supporting Information

S1 Table Increase in transcription of proinflammatory molecules in cochlea of MCMV infected mice. RNA was isolated from the cochlea of mock infected and MCMV infected mice at the indicated postnatal day (PNd) of life and 50ng reverse transcribed and assayed using PCR arrays as described in Materials and Methods. Number of animals are listed in parentheses and PCR results compared to RNA prepared from cochlea of mock infected animals (PNd4 n = 16; PNd8 n = 16; Pnd16 n = 18). Results are expressed as fold increase over signal from cochlea obtained in mock infected animals with statistical measures of differences shown as *p* value.
(TIF)

S1 Fig Histological characteristics of the Organ of Corti in MCMV (A, B,C) and Mock (D,E,F) infected mice. Newborn mice infected with 200 PFU of tissue culture derived Smith strain MCMV, or mock infected, were sacrificed on postnatal day (PNd) 11 and after perfusion with PBS and fixation, the inner ears were removed, decalcified and embedded in paraffin as described in Materials and Methods. After sectioning, de-paraffinization, sections were stained with hematoxylin and eosin (H&E) and photographed (x40). Panel C and F represent digitally enlarged images of Panel B and E respectively.
(TIF)

S2 Fig MCMV infection in cells of temporal bone marrow. After extensive perfusion, temporal bones from mice infected with 200PFU Smith MCMV were harvested on PNd7, fixed and embedded in paraffin as described in Materials and Methods. Sections were cut and stained with H&E (Panels A, B; 100x, scale bar 50um). Boxed areas demonstrated large cell with characteristics of MCMV infected cell. Other sections were reacted with anti-MCMV IE-1 antibodies and developed with HRP anti-mouse IgG followed with diaminobenzidine to produce brown color in infected cells (Panel C; 40x, scale bar 100um). Scale bars shown in lower right corner.
(TIF)

S3 Fig MCMV infection of marginal cell of stria vascularis. Cochlea from mock infected and MCMV infected mice were harvested on PNd7, decalcified, fixed and embedded in paraffin as described in Materials and Methods. MCMV infected cells were stained with anti-IE-1 antibodies and then developed with HRP anti-mouse IgG and diaminobenzidine (brown). This section reveals infected cell in marginal layer of stria vascularis. Panel A at 20x (scale bar at 100um) and panel B at 100x (scale bar at 50nm). Panel C is digitally enlarged image of panel B.
(TIF)

S4 Fig Distortion product otoacoustic emission (DPOAE) testing. DPOAE revealed a significant differences at only one frequency between mock infected (**○**; n = 25) and MCMV infected (■; n = 25) mice. Mice were tested at PNd42 as described in Materials and Methods. (*) indicates significant difference in response at 8 kHz.
(TIF)

S5 Fig Histological findings in cochlea in mock and MCMV infected mice with normal hearing and hearing loss. After extensive perfusion, cochlea from mice between PNd42-60 were harvested, decalcified, fixed and embedded in paraffin as described in Materials and Methods. Following sectioning and staining with H&E, individual sections were viewed by blinded observer with experience in cochlear histology. Sections from 10 different cochlea were submitted for review and four representative sections are presented this figure. Samples 127 and 133 were from mock infected animals and samples 147 and 211 were from infected animals. The ABR thresholds for these ears are displayed at the bottom of each panel.
(TIF)

S6 Fig Loss of spiral ganglion neurons in MCMV infected mice. After extensive perfusion, cochlea from mock infected and MCMV infected mice were harvested on PNd7, decalcified, fixed and embedded in paraffin as described in Materials and Methods. Following sectioning, tissue was reacted with anti-Tuji 1 followed by HRP conjugated anti-mouse IgG and developed with diaminobenzidine(brown). Note the decreased density of Tuji 1+ cells in section from infected mouse (100x magnification with 50um scale bar lower right corner).
(TIF)

## References

[1] Boppana S, Britt WJ (2013) Synopsis of clinical aspects of human cytomegalovirus disease In: Reddehasse M, editor. Cytomegaloviruses. London: Cassister.

[2] Mocarski ES, Tan Courcelle C. (2001) Cytomegaloviruses and their replication In: Howley DMKaPM, editor. Fields Virology. 4th ed. Philadelphia: Lippincott Williams and Wilkins pp. 2629–2673.

[3] Dreher AM, Arora N, Fowler KB, Novak Z, Britt WJ, et al (2014) Spectrum of Disease and Outcome in Children with Symptomatic Congenital Cytomegalovirus Infection. J Pediatr 164: 855–859. 10.1016/j.jpeds.2013.12.007 24433826 PMC3982912

[4] Dahle AJ, Fowler KB, Wright JD, Boppana SB, Britt WJ, et al (2000) Longitudinal investigation of hearing disorders in children with congenital cytomegalovirus. Journal of the American Academy of Audiology 11: 283–290. 10821506

[5] Fowler KB (2013) Congenital cytomegalovirus infection: audiologic outcome. Clin Infect Dis 57 Suppl 4: S182–184. 10.1093/cid/cit609 24257423 PMC3836573

[6] Morton CC, Nance WE (2006) Newborn hearing screening—a silent revolution. N Engl J Med 354: 2151–2164. 16707752 10.1056/NEJMra050700

[7] Yamamoto AY, Mussi-Pinhata MM, Isaac MDL, Amaral FR, Carvalheiro CG, et al (2011) Congenital cytomegalovirus infection as a cause of sensorineural hearing loss in a highly seropositive population. Ped Infect Dis J 30: 1043–1046. 10.1097/INF.0b013e31822d9640 21814153 PMC3222783

[8] Dar L, Pati SK, Patro AR, Deorari AK, Rai S, et al (2008) Congenital cytomegalovirus infection in a highly seropositive semi-urban population in India. Pediatr Infect Dis J 27: 841–843. 10.1097/INF.0b013e3181723d55 18645544

[9] Manicklal S, Emery VC, Lazzarotto T, Boppana SB, Gupta RK (2013) The "silent" global burden of congenital cytomegalovirus. Clin Microbiol Rev 26: 86–102. 10.1128/CMR.00062-12 23297260 PMC3553672

[10] Kimberlin DW, Lin CY, Sanchez PJ, Demmler GJ, Dankner W, et al (2003) Effect of ganciclovir therapy on hearing in symptomatic congenital cytomegalovirus disease involving the central nervous system: a randomized, controlled trial.[see comment]. Journal of Pediatrics 143: 16–25. 12915819 10.1016/s0022-3476(03)00192-6

[11] Boppana SB, Britt WJ (2014) Recent approaches and strategies in the generation of antihuman cytomegalovirus vaccines. Methods Mol Biol 1119: 311–348. 10.1007/978-1-62703-788-4_17 24639230

[12] Fowler KB, McCollister FP, Dahle AJ, Boppana S, Britt WJ, et al (1997) Progressive and fluctuating sensorineural hearing loss in children with asymptomatic congenital cytomegalovirus infection. Journal of Pediatrics 130: 624–630. 9108862 10.1016/s0022-3476(97)70248-8

[13] Davis LE, Johnsson LG, Kornfeld M (1981) Cytomegalovirus labyrinthitis in an infant: morphological, virological, and immunofluorescent studies. J Neuropathol Exp Neurol 40: 9–19. 6259297

[14] Boppana S, Britt W (2006) Cytomegalovirus In: Newton VE, Vallely PJ, editors. Infection and Hearing Impairment. Sussex, UK: John Wiley and Sons pp. 67–93.

[15] Harris JP, Fan JT, Keithley EM (1990) Immunologic responses in experimental cytomegalovirus labyrinthitis. Am J Otolaryngol 11: 304–308. 2176064 10.1016/0196-0709(90)90059-5

[16] Woolf NK, Koehrn FJ, Harris JP, Richman DD (1989) Congenital cytomegalovirus labyrinthitis and sensorineural hearing loss in guinea pigs. J Infect Dis 160: 929–937. 2555420 10.1093/infdis/160.6.929

[17] Park AH, Gifford T, Schleiss MR, Dahlstrom L, Chase S, et al (2010) Development of cytomegalovirus-mediated sensorineural hearing loss in a Guinea pig model. Arch Otolaryngol Head Neck Surg 136: 48–53. 10.1001/archoto.2009.210 20083778

[18] Wang Y, Patel R, Ren C, Taggart MG, Firpo MA, et al (2013) A comparison of different murine models for cytomegalovirus-induced sensorineural hearing loss. Laryngoscope 123: 2801–2806. 10.1002/lary.24090 23616191

[19] Schachtele SJ, Mutnal MB, Schleiss MR, Lokensgard JR Cytomegalovirus-induced sensorineural hearing loss with persistent cochlear inflammation in neonatal mice. J Neurovirol 17: 201–211. 10.1007/s13365-011-0024-7 21416394 PMC3098308

[20] Juanjuan C, Yan F, Li C, Haizhi L, Ling W, et al (2011) Murine model for congenital CMV infection and hearing impairment. Virol J 8: 70 10.1186/1743-422X-8-70 21320351 PMC3045346

[21] Li L, Kosugi I, Han GP, Kawasaki H, Arai Y, et al. (2008) Induction of cytomegalovirus-infected labyrinthitis in newborn mice by lipopolysaccharide: a model for hearing loss in congenital CMV infection. Lab Invest. 10.1038/labinvest.2008.39 18475257

[22] Katano H, Sato Y, Tsutsui Y, Sata T, Maeda A, et al (2007) Pathogenesis of cytomegalovirus-associated labyrinthitis in a guinea pig model. Microbes Infect 9: 183–191. 17208485 10.1016/j.micinf.2006.11.004

[23] Koontz T, Bralic M, Tomac J, Pernjak-Pugel E, Bantug G, et al (2008) Altered development of the brain after focal herpesvirus infection of the central nervous system. J Exp Med 205: 423–435. 10.1084/jem.20071489 18268036 PMC2271002

[24] Kosmac K, Bantug GR, Pugel EP, Cekinovic D, Jonjic S, et al (2013) Glucocortiocoid Treatment of MCMV Infected Newborn Mice Attenuates CNS Inflammation and Limits Deficits in Cerebellar Development. PLoS Pathog 9: e1003200 10.1371/journal.ppat.1003200 23505367 PMC3591306

[25] Jordan S, Krause J, Prager A, Mitrovic M, Jonjic S, et al (2011) Virus progeny of murine cytomegalovirus bacterial artificial chromosome pSM3fr show reduced growth in salivary Glands due to a fixed mutation of MCK-2. J Virol 85: 10346–10353. 10.1128/JVI.00545-11 21813614 PMC3196435

[26] Zheng QY, Johnson KR, Erway LC (1999) Assessment of hearing in 80 inbred strains of mice by ABR threshold analyses. Hear Res 130: 94–107. 10320101 10.1016/s0378-5955(99)00003-9 PMC2855304

[27] Willott JF, Turner JG, Carlson S, Ding D, Seegers Bross L, et al (1998) The BALB/c mouse as an animal model for progressive sensorineural hearing loss. Hear Res 115: 162–174. 9472745 10.1016/s0378-5955(97)00189-5

[28] Brandt CT, Caye-Thomasen P, Lund SP, Worsoe L, Ostergaard C, et al (2006) Hearing loss and cochlear damage in experimental pneumococcal meningitis, with special reference to the role of neutrophil granulocytes. Neurobiol Dis 23: 300–311. 16798006 10.1016/j.nbd.2006.03.006

[29] Klein M, Koedel U, Pfister HW, Kastenbauer S (2003) Morphological correlates of acute and permanent hearing loss during experimental pneumococcal meningitis. Brain Pathol 13: 123–132. 12744466 10.1111/j.1750-3639.2003.tb00012.x PMC8095810

[30] Nadol JB Jr., Hsu WC (1991) Histopathologic correlation of spiral ganglion cell count and new bone formation in the cochlea following meningogenic labyrinthitis and deafness. Ann Otol Rhinol Laryngol 100: 712–716. 1952661 10.1177/000348949110000904

[31] Boppana SB, Britt WJ (2006) Cytomegalovirus In: Newton VE, Vallely PJ, editors. Infection and Hearing Impairment. West Sussex, England: Wiley pp. 67–91.

[32] Teissier N, Delezoide AL, Mas AE, Khung-Savatovsky S, Bessieres B, et al (2011) Inner ear lesions in congenital cytomegalovirus infection of human fetuses. Acta Neuropathol 122: 763–774. 10.1007/s00401-011-0895-y 22033878

[33] Schleiss MR (2006) Nonprimate models of congenital cytomegalovirus (CMV) infection: gaining insight into pathogenesis and prevention of disease in newborns. ILAR J 47: 65–72. 16391432 10.1093/ilar.47.1.65

[34] Alam SA, Robinson BK, Huang J, Green SH (2007) Prosurvival and proapoptotic intracellular signaling in rat spiral ganglion neurons in vivo after the loss of hair cells. J Comp Neurol 503: 832–852. 17570507 10.1002/cne.21430

[35] Trune DR (1982) Influence of neonatal cochlear removal on the development of mouse cochlear nucleus: I. Number, size, and density of its neurons. J Comp Neurol 209: 409–424. 7130465 10.1002/cne.902090410

[36] Webster DB (1983) A critical period during postnatal auditory development of mice. Int J Pediatr Otorhinolaryngol 6: 107–118. 6662618 10.1016/s0165-5876(83)80111-6

[37] Harris JA, Rubel EW (2006) Afferent regulation of neuron number in the cochlear nucleus: cellular and molecular analyses of a critical period. Hear Res 216–217: 127–137. 10.1016/j.heares.2006.03.016 16874907

[38] Rubel EW, Fritzsch B (2002) Auditory system development: primary auditory neurons and their targets. Annu Rev Neurosci 25: 51–101. 12052904 10.1146/annurev.neuro.25.112701.142849

[39] Tierney TS, Russell FA, Moore DR (1997) Susceptibility of developing cochlear nucleus neurons to deafferentation-induced death abruptly ends just before the onset of hearing. J Comp Neurol 378: 295–306. 9120067 10.1002/(sici)1096-9861(19970210)378:2<295::aid-cne11>3.0.co;2-r

[40] Harris JA, Iguchi F, Seidl AH, Lurie DI, Rubel EW (2008) Afferent deprivation elicits a transcriptional response associated with neuronal survival after a critical period in the mouse cochlear nucleus. J Neurosci 28: 10990–11002. 10.1523/JNEUROSCI.2697-08.2008 18945907 PMC2585504

[41] Mostafapour SP, Del Puerto NM, Rubel EW (2002) bcl-2 Overexpression eliminates deprivation-induced cell death of brainstem auditory neurons. J Neurosci 22: 4670–4674. 12040073 10.1523/JNEUROSCI.22-11-04670.2002 PMC6758828

[42] Leake PA, Hradek GT, Hetherington AM, Stakhovskaya O (2011) Brain-derived neurotrophic factor promotes cochlear spiral ganglion cell survival and function in deafened, developing cats. J Comp Neurol 519: 1526–1545. 10.1002/cne.22582 21452221 PMC3079794

[43] Miller JM, Le Prell CG, Prieskorn DM, Wys NL, Altschuler RA (2007) Delayed neurotrophin treatment following deafness rescues spiral ganglion cells from death and promotes regrowth of auditory nerve peripheral processes: effects of brain-derived neurotrophic factor and fibroblast growth factor. J Neurosci Res 85: 1959–1969. 17492794 10.1002/jnr.21320

[44] Ramekers D, Versnel H, Grolman W, Klis SF (2012) Neurotrophins and their role in the cochlea. Hear Res 288: 19–33. 10.1016/j.heares.2012.03.002 22465680

[45] Zhai SQ, Guo W, Hu YY, Yu N, Chen Q, et al (2011) Protective effects of brain-derived neurotrophic factor on the noise-damaged cochlear spiral ganglion. J Laryngol Otol 125: 449–454. 10.1017/S0022215110002112 21078216

[46] Nickel R, Forge A (2008) Gap junctions and connexins in the inner ear: their roles in homeostasis and deafness. Curr Opin Otolaryngol Head Neck Surg 16: 452–457. 10.1097/MOO.0b013e32830e20b0 18797288

[47] Ciuman RR (2009) Stria vascularis and vestibular dark cells: characterisation of main structures responsible for inner-ear homeostasis, and their pathophysiological relations. J Laryngol Otol 123: 151–162. 10.1017/S0022215108002624 18570690

[48] Kennedy HJ (2012) New developments in understanding the mechanisms and function of spontaneous electrical activity in the developing mammalian auditory system. J Assoc Res Otolaryngol 13: 437–445. 10.1007/s10162-012-0325-4 22526733 PMC3387308

[49] Tritsch NX, Bergles DE (2010) Developmental regulation of spontaneous activity in the Mammalian cochlea. J Neurosci 30: 1539–1550. 10.1523/JNEUROSCI.3875-09.2010 20107081 PMC2814371

[50] Martin GK, Stagner BB, Lonsbury-Martin BL (2006) Assessment of cochlear function in mice: distortion-product otoacoustic emissions. Curr Protoc Neurosci Chapter 8: Unit8 21C. 10.1002/0471142301.ns0821cs34 18428646

[51] Willott JF (2006) Measurement of the auditory brainstem response (ABR) to study auditory sensitivity in mice. Curr Protoc Neurosci Chapter 8: Unit8 21B. 10.1002/0471142301.ns0821bs34 18428645

