# Supplementary figures and images for: Murine CMV-Induced Hearing Loss Is Associated with Inner Ear Inflammation and Loss of Spiral Ganglia Neurons
Source: PLoS Pathog. 2015 Apr 13;11(4):e1004774. doi: 10.1371/journal.ppat.1004774 (PMC4395355; doi:10.1371/journal.ppat.1004774)

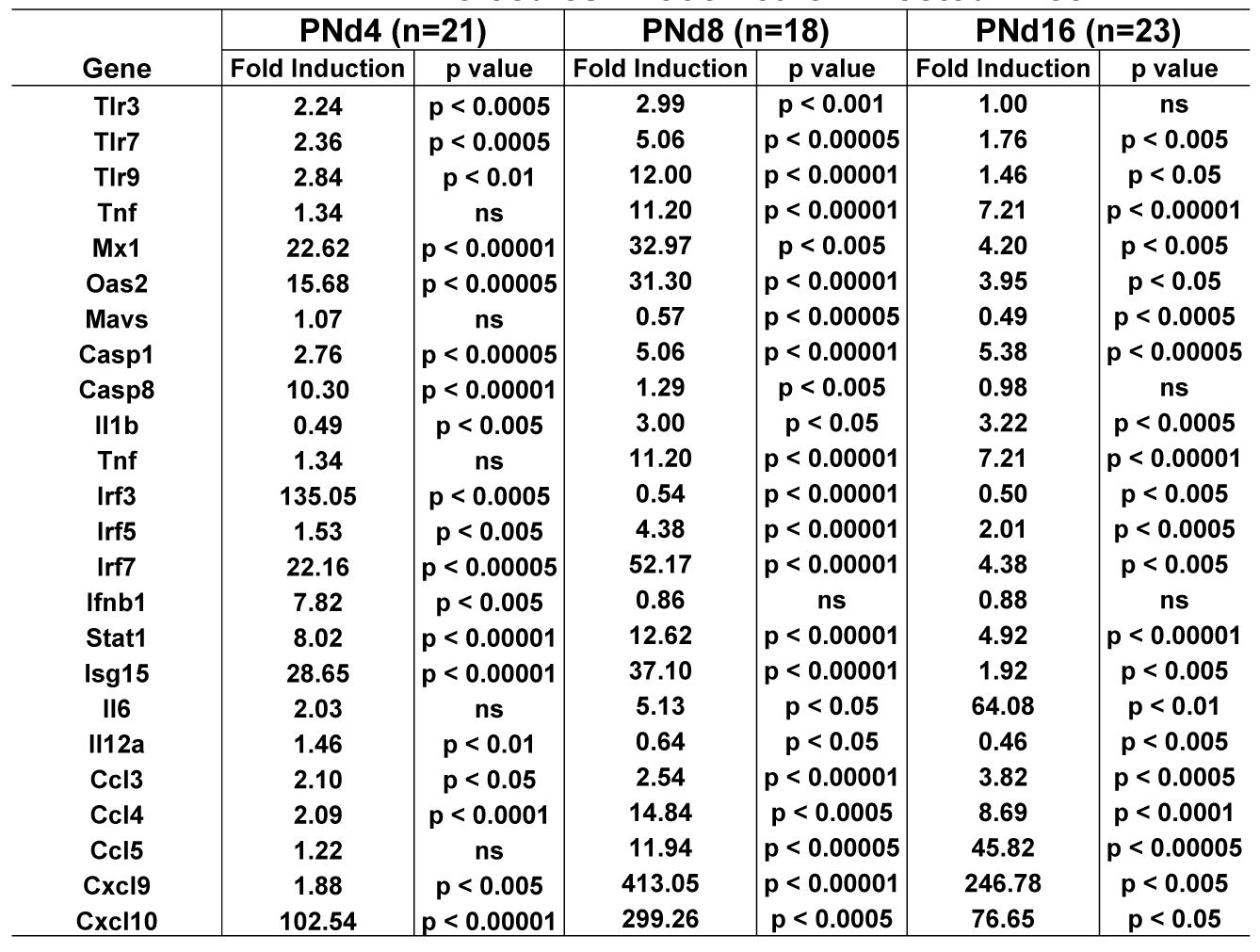

Supplement: S1 Table — RNA was isolated from the cochlea of mock infected and MCMV infected mice at the indicated postnatal day (PNd) of life and 50ng reverse transcribed and assayed using PCR arrays as described in Materials and Methods. Number of animals are listed in parentheses and PCR results compared to RNA prepared from cochlea of mock infected animals (PNd4 n = 16; PNd8 n = 16; Pnd16 n = 18). Results are expressed as fold increase over signal from cochlea obtained in mock infected animals with statistical measures of differences shown as p value. (TIF) [file ppat.1004774.s001.tif]

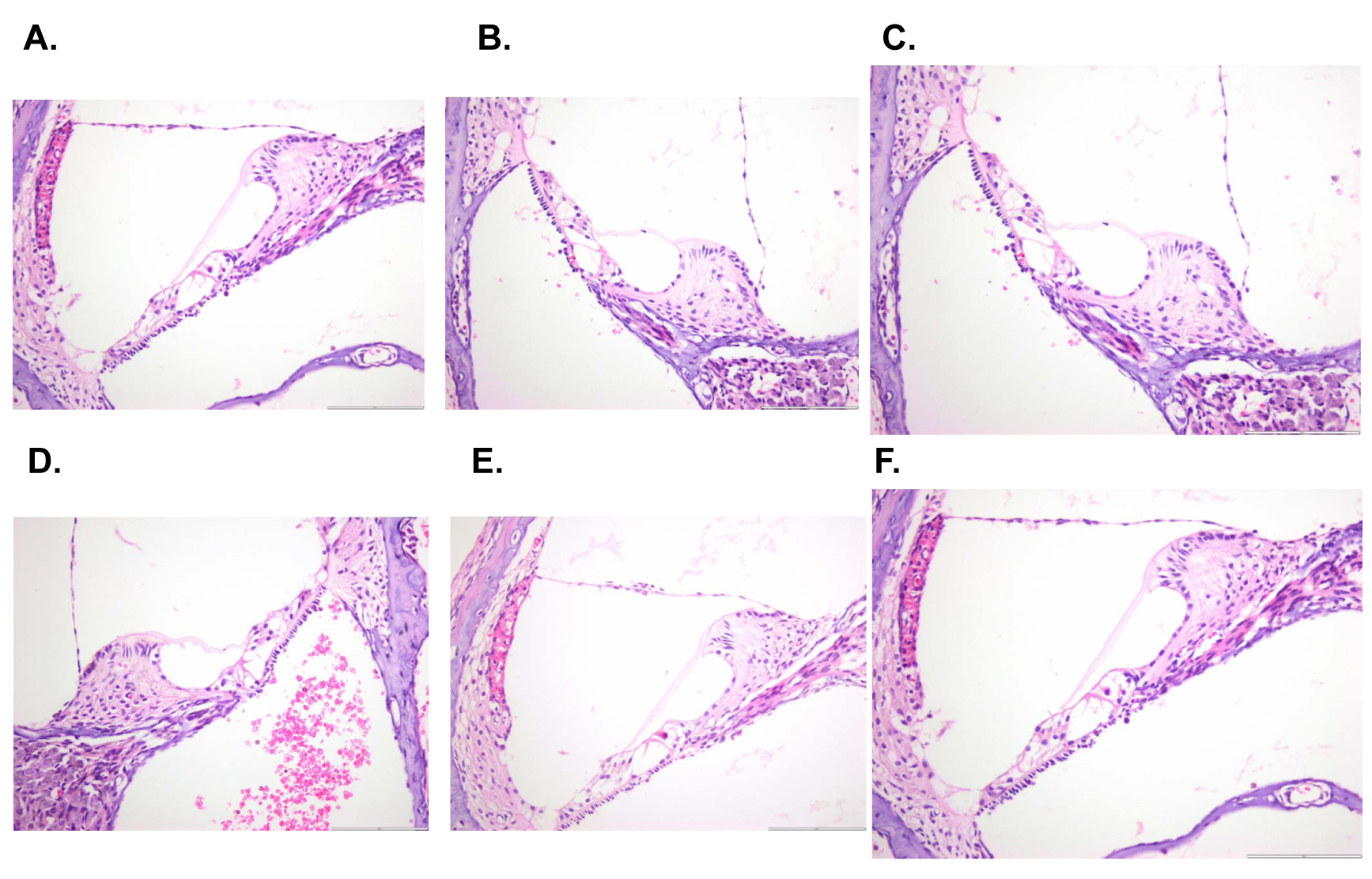

Supplement: S1 Fig — Newborn mice infected with 200 PFU of tissue culture derived Smith strain MCMV, or mock infected, were sacrificed on postnatal day (PNd) 11 and after perfusion with PBS and fixation, the inner ears were removed, decalcified and embedded in paraffin as described in Materials and Methods. After sectioning, de-paraffinization, sections were stained with hematoxylin and eosin (H&E) and photographed (x40). Panel C and F represent digitally enlarged images of Panel B and E respectively. (TIF) [file ppat.1004774.s002.tif]

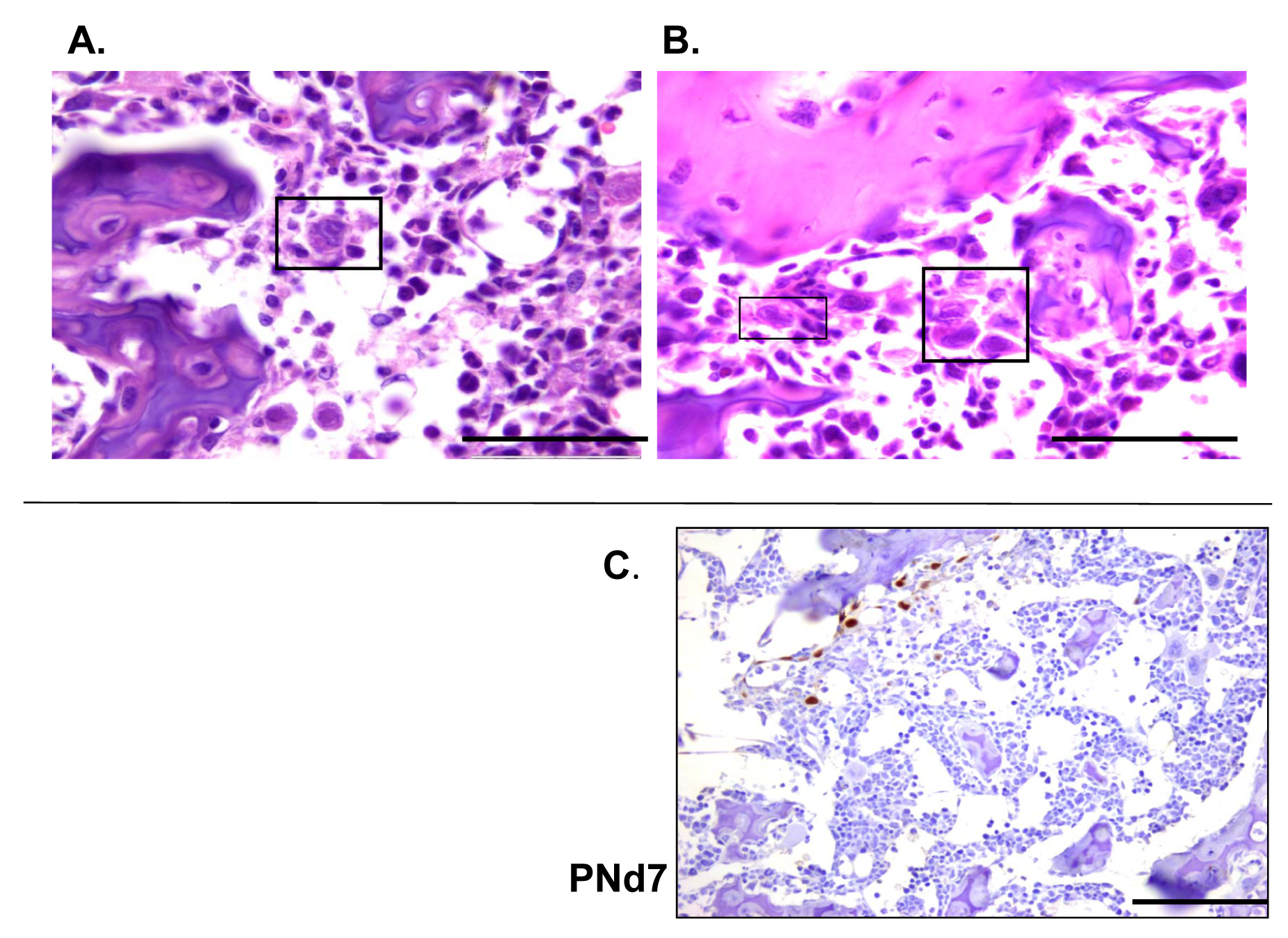

Supplement: S2 Fig — After extensive perfusion, temporal bones from mice infected with 200PFU Smith MCMV were harvested on PNd7, fixed and embedded in paraffin as described in Materials and Methods. Sections were cut and stained with H&E (Panels A, B; 100x, scale bar 50um). Boxed areas demonstrated large cell with characteristics of MCMV infected cell. Other sections were reacted with anti-MCMV IE-1 antibodies and developed with HRP anti-mouse IgG followed with diaminobenzidine to produce brown color in infected cells (Panel C; 40x, scale bar 100um). Scale bars shown in lower right corner. (TIF) [file ppat.1004774.s003.tif]

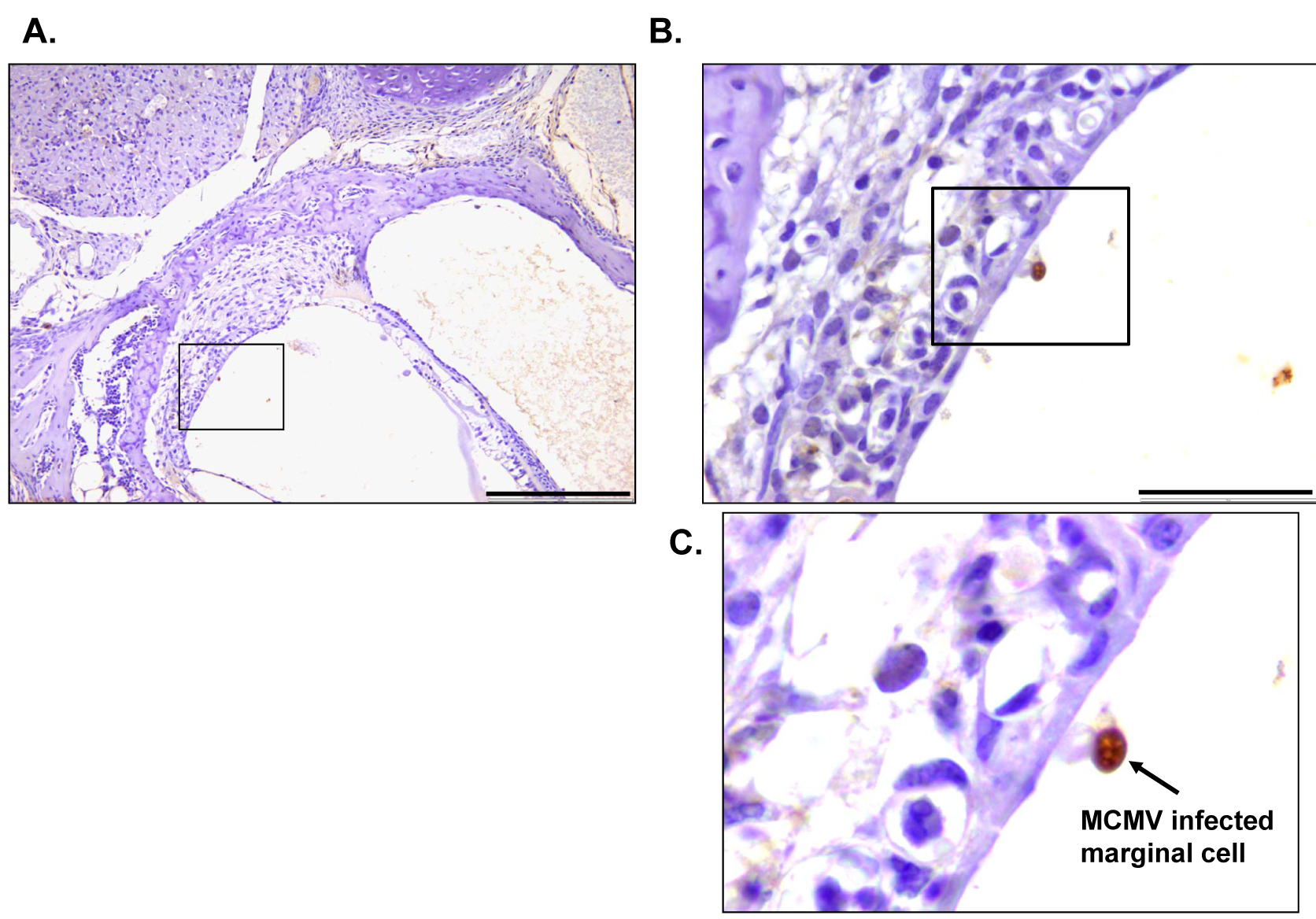

Supplement: S3 Fig — Cochlea from mock infected and MCMV infected mice were harvested on PNd7, decalcified, fixed and embedded in paraffin as described in Materials and Methods. MCMV infected cells were stained with anti-IE-1 antibodies and then developed with HRP anti-mouse IgG and diaminobenzidine (brown). This section reveals infected cell in marginal layer of stria vascularis. Panel A at 20x (scale bar at 100um) and panel B at 100x (scale bar at 50nm). Panel C is digitally enlarged image of panel B. (TIF) [file ppat.1004774.s004.tif]

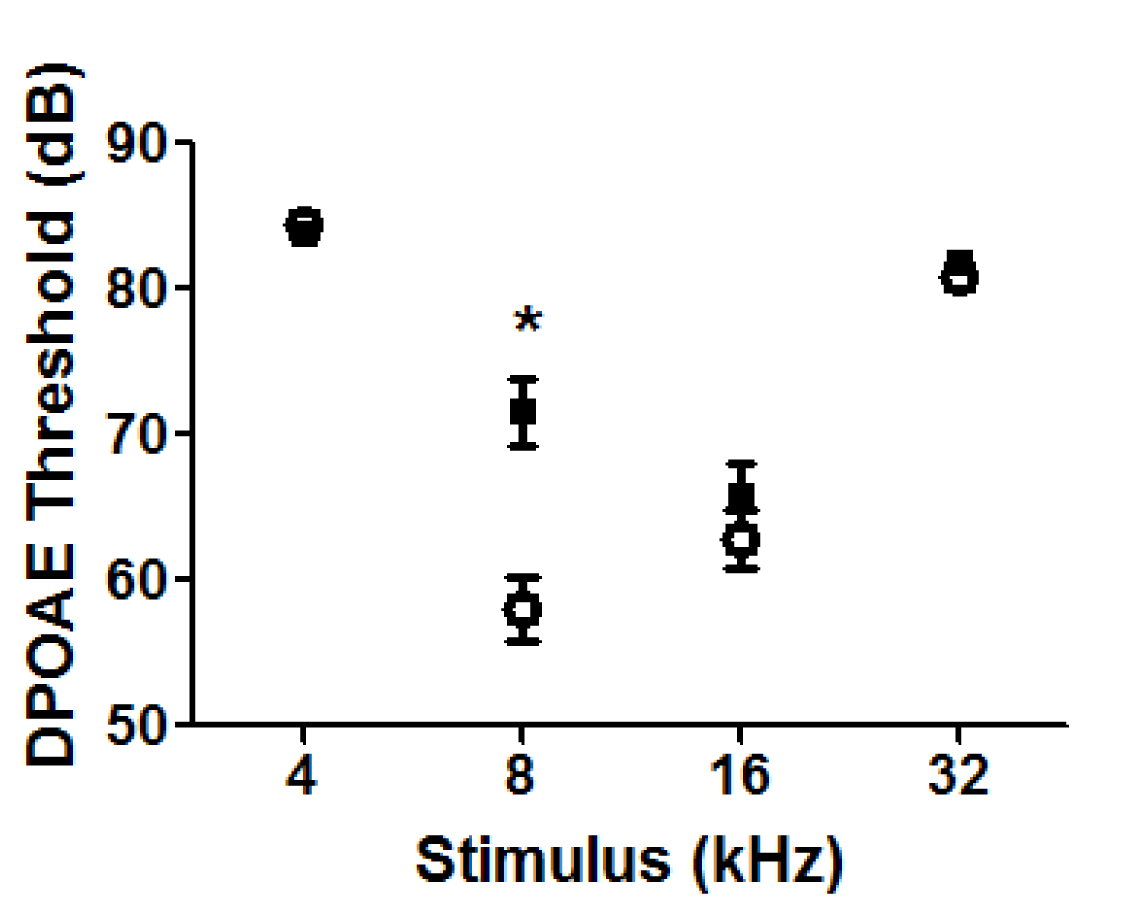

Supplement: S4 Fig — DPOAE revealed a significant differences at only one frequency between mock infected (○; n = 25) and MCMV infected (■; n = 25) mice. Mice were tested at PNd42 as described in Materials and Methods. (*) indicates significant difference in response at 8 kHz. (TIF) [file ppat.1004774.s005.tif]

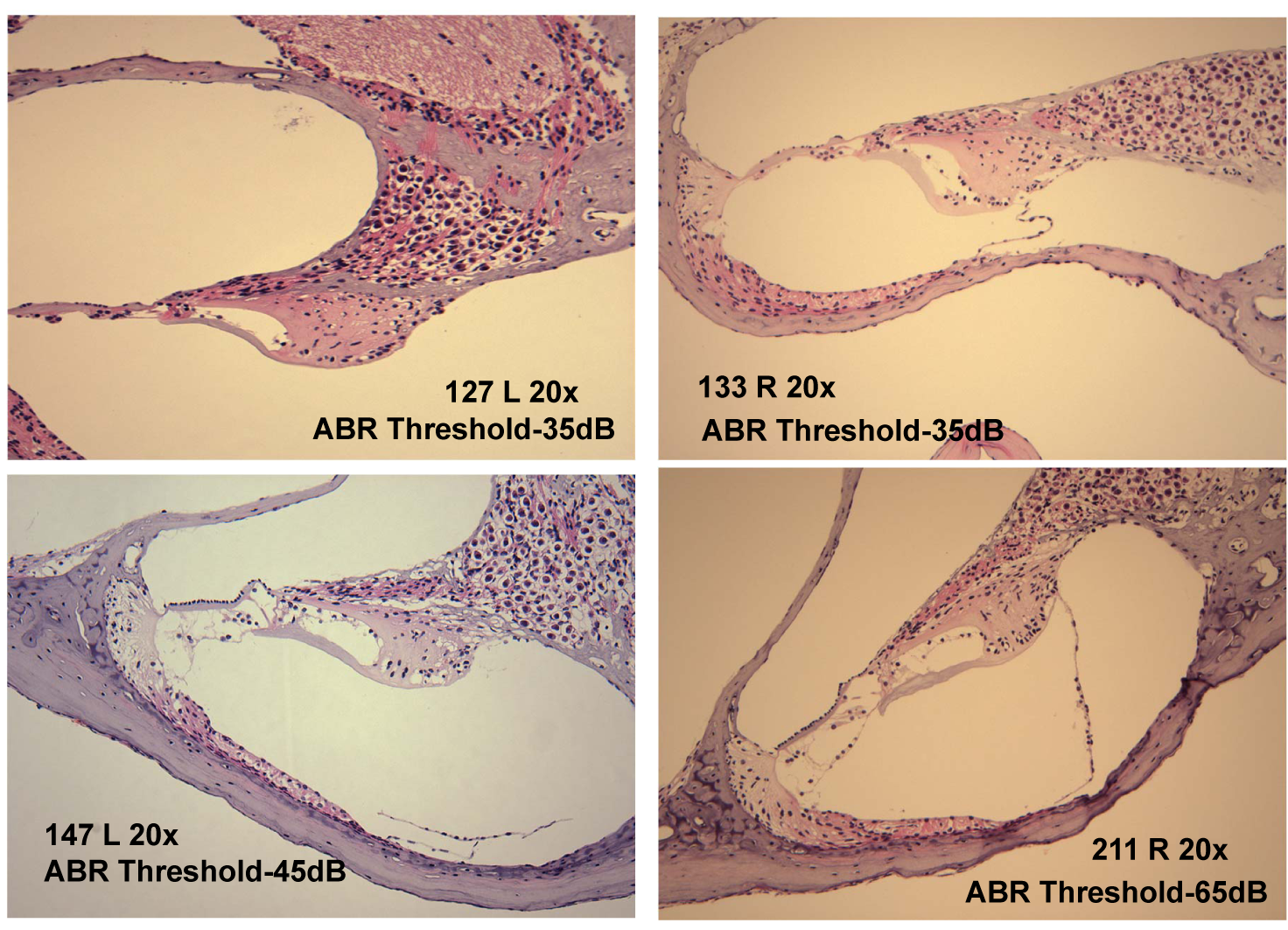

Supplement: S5 Fig — After extensive perfusion, cochlea from mice between PNd42-60 were harvested, decalcified, fixed and embedded in paraffin as described in Materials and Methods. Following sectioning and staining with H&E, individual sections were viewed by blinded observer with experience in cochlear histology. Sections from 10 different cochlea were submitted for review and four representative sections are presented this figure. Samples 127 and 133 were from mock infected animals and samples 147 and 211 were from infected animals. The ABR thresholds for these ears are displayed at the bottom of each panel. (TIF) [file ppat.1004774.s006.tif]

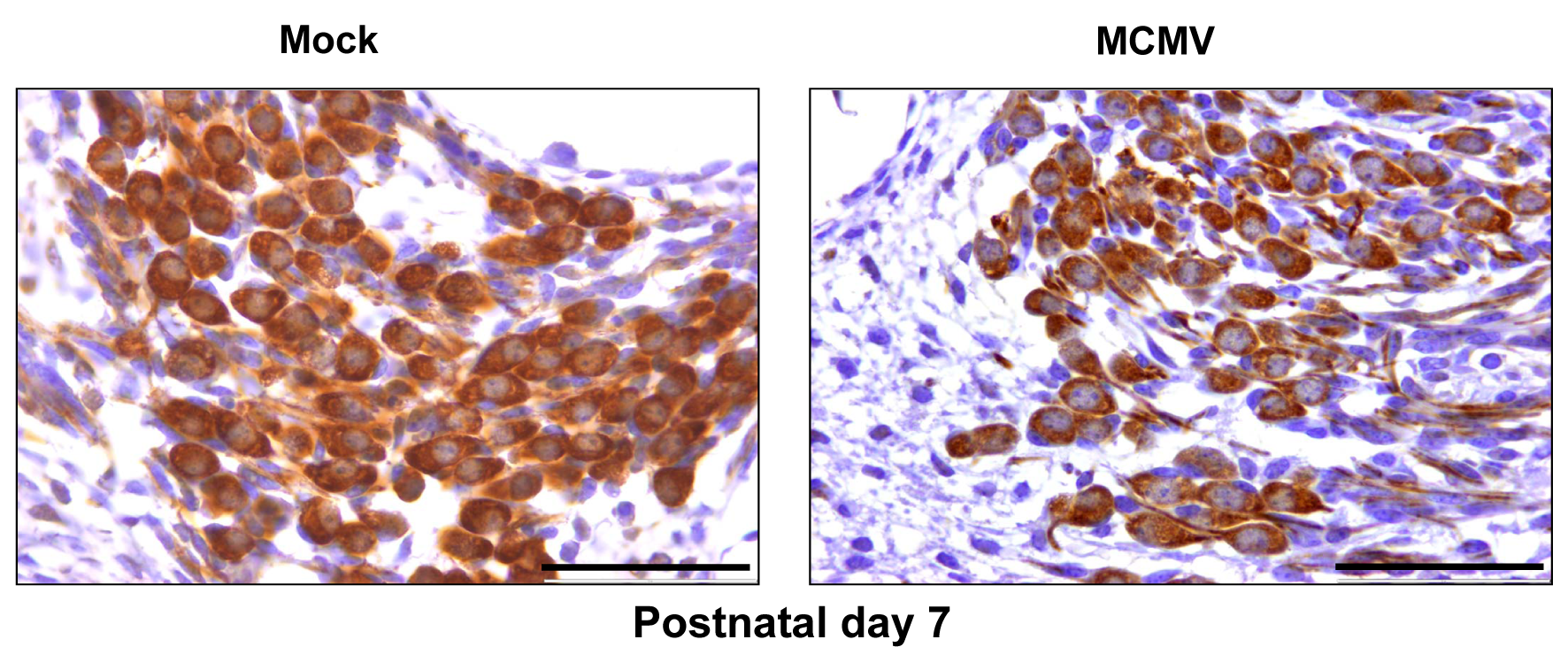

Supplement: S6 Fig — After extensive perfusion, cochlea from mock infected and MCMV infected mice were harvested on PNd7, decalcified, fixed and embedded in paraffin as described in Materials and Methods. Following sectioning, tissue was reacted with anti-Tuji 1 followed by HRP conjugated anti-mouse IgG and developed with diaminobenzidine(brown). Note the decreased density of Tuji 1+ cells in section from infected mouse (100x magnification with 50um scale bar lower right corner). (TIF) [file ppat.1004774.s007.tif]
